# ERBB and P‐glycoprotein inhibitors break resistance in relapsed neuroblastoma models through P‐glycoprotein

**DOI:** 10.1002/1878-0261.13318

**Published:** 2022-11-05

**Authors:** Lisa Rösch, Sonja Herter, Sara Najafi, Johannes Ridinger, Heike Peterziel, Jindrich Cinatl, David T. W. Jones, Martin Michaelis, Olaf Witt, Ina Oehme

**Affiliations:** ^1^ Hopp Children's Cancer Center Heidelberg (KiTZ) Germany; ^2^ Clinical Cooperation Unit Pediatric Oncology German Cancer Research Center (DKFZ) and German Cancer Consortium (DKTK) Heidelberg Germany; ^3^ Faculty of Biosciences University of Heidelberg Germany; ^4^ Department of Pediatric Oncology, Hematology and Immunology University Hospital Heidelberg Germany; ^5^ Institute for Medical Virology Goethe University Hospital Frankfurt am Main Germany; ^6^ Division of Pediatric Glioma Research German Cancer Research Center (DKFZ) Heidelberg Germany; ^7^ School of Biosciences University of Kent Canterbury UK

**Keywords:** apoptotic cell death, chemotherapy resistance, off‐target, pediatric patient samples, precision medicine, zebrafish xenograft model

## Abstract

Chemotherapy resistance is a persistent clinical problem in relapsed high‐risk neuroblastomas. We tested a panel of 15 drugs for sensitization of neuroblastoma cells to the conventional chemotherapeutic vincristine, identifying tariquidar, an inhibitor of the transmembrane pump P‐glycoprotein (P‐gp/*ABCB1*), and the ERBB family inhibitor afatinib as the top resistance breakers. Both compounds were efficient in sensitizing neuroblastoma cells to vincristine in trypan blue exclusion assays and in inducing apoptotic cell death. The evaluation of ERBB signaling revealed no functional inhibition, that is, dephosphorylation of the downstream pathways upon afatinib treatment but direct off‐target interference with P‐gp function. Depletion of *ABCB1*, but not *ERRB4*, sensitized cells to vincristine treatment. P‐gp inhibition substantially broke vincristine resistance *in vitro* and *in vivo* (zebrafish embryo xenograft). The analysis of gene expression datasets of more than 50 different neuroblastoma cell lines (primary and relapsed) and more than 160 neuroblastoma patient samples from the pediatric precision medicine platform INFORM (Individualized Therapy For Relapsed Malignancies in Childhood) confirmed a pivotal role of P‐gp specifically in neuroblastoma resistance at relapse, while the ERBB family appears to play a minor part.

## Graphical abstract

Chemotherapy resistance is a clinical problem in relapsed neuroblastomas (NBs). With a vincristine (VCR, yellow symbol) resistant model (A), we identified tariquidar (green), an inhibitor of the efflux pump P‐gp/*ABCB1*, and afatinib (orange), an ERBB inhibitor, as resistance breaker (B). The comprehensive analysis of *ERBB4* and P‐gp/*ABCB1* expression and function (C), revealed that both drugs act through P‐gp inhibition (D).

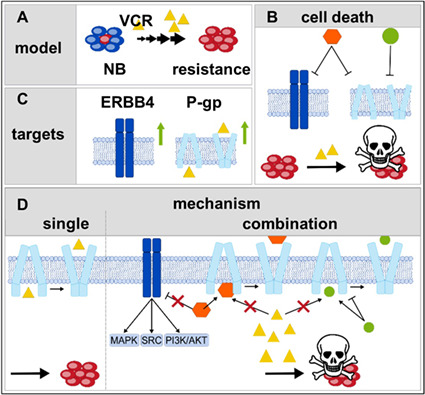

**Abbreviations**
4ICD: cleaved, soluble intracellular domain of ERBB4
ABCB1: ATP binding cassette subfamily B member 1
AUC: area under the curve
BID: BH3‐interacting domain death agonist
DMSO: dimethylsulfoxide
EGFR: epidermal growth factor receptor
ERBB: erythroblastic leukemia viral oncogene homolog
FCS: fetal calf serum
GAPDH: glyceraldehyde‐3‐phosphate dehydrogenase
INFORM: individualized therapy for relapsed malignancies in childhood
MAPK: mitogen‐activated protein kinase
PARP: poly(ADP‐ribose) polymerase
PI3K: phosphoinositide 3‐kinase
tBID: truncated BID
VCR: vincristine

## 1 Introduction

Neuroblastoma is a pediatric tumor of the sympathetic nervous system originating from neural crest cells [1]. Neuroblastoma cases are stratified into different risk groups, the outcomes of which differ substantially [2]. Patients included in the low‐ and intermediate‐risk category have very good chances of survival, and at times, their tumors even regress spontaneously [3]. High‐risk neuroblastoma includes patients with an amplification of the MYCN gene or patients with metastatic disease and > 18 months of age at the time of diagnosis [2]. Patients with high‐risk neuroblastoma still have an overall survival rate below 50% [4]. One subgroup with a particularly dismal outcome is high‐risk patients after relapse, with a median post‐relapse survival of 4.5 months and a 5‐year overall survival under 10% [5]. This patient group with highly aggressive and often therapy‐resistant tumors is in great need of novel treatment strategies to overcome or circumvent therapy resistance.

A central question in the field of cancer biology is how tumor cells develop an invasive and drug‐resistant phenotype. Unraveling the underlying mechanisms helps to develop new compounds or to optimize approved drug combinations to exploit specific vulnerabilities. One possibility to target relapsed tumors, in particular, is by a ‘one‐two punch’ model. This model proposes that changes in the tumor cells brought on by chemotherapy (first punch) lead to novel vulnerabilities that may be specifically targeted (second punch) [6]. We utilized this model of resistance by comparing neuroblastoma cell lines derived before and after the patient received chemotherapy as well as cell lines that mirror this by having been exposed to increasing concentrations of the common chemotherapeutic vincristine (VCR), thereby becoming resistant.

The ERBB family of receptor tyrosine kinases is well known for its link to cancer [7]. The family consists of four members, EGFR (ERBB1), ERBB2 (HER2), ERBB3, and ERBB4, which form homo and/or heterodimers upon ligand binding, leading to autophosphorylation and activation of downstream signaling [8]. Downstream pathways include the PI3K/AKT, SRC, and MAPK signaling pathways and lead to increased proliferation and tumor cell survival [9, 10, 11].

When ERBB3 was first discovered, it was considered an inactive pseudokinase due to critical amino acid substitutions involved in ATP binding [12, 13, 14]. Later, however, it was shown to have a weak capability of trans‐autophosphorylation [15]. This phosphorylation capability is more than 1000‐fold weaker than that of EGFR, showing ERBB3 to be a kinase impaired but not a kinase‐dead receptor. For efficient signaling, ERBB3 requires heterodimerization with another member of the ERBB family, with ERBB2 being the preferred interaction partner [16]. This leads to a very active ERBB2‐ERBB3 heterodimer with strong proliferative and pro‐oncogenic properties [17]. ERBB3 is essential for the correct migration of Schwann cell precursors and mesenchymal stem cells during development [18, 19]. Neuroblastomas with a mesenchymal phenotype are considered high risk and more resistant to chemotherapy [20], making ERBB3 an interesting target in neuroblastoma research.

ERBB4 is a special case within the ERBB family, as its role in cancer development is diverse, ranging from tumorigenic to cell death inducing [11, 21, 22, 23]. Some studies have shown that high ERBB4 levels can even be correlated with a positive prognosis [24]. ERBB4 has four isoforms, two of which can be cleaved by TACE and γ‐secretase to yield a soluble intracellular domain (4ICD) [25]. 4ICD interacts with YAP and can translocate to the nucleus, where it directly influences transcription, for example, of genes involved in cell migration [26]. It can also translocate to the mitochondria and directly induce cell death [22].

P‐glycoprotein (P‐gp/*ABCB1*) is a transmembrane transporter of the ABC (ATP binding cassette) transporter family, which consists of over 40 members in seven subfamilies [27]. The involvement of P‐gp in chemotherapy resistance is well documented [28]. P‐gp efficiently transports its substrates out of the cell, which includes a wide range of both conventional chemotherapeutics and targeted treatments [28, 29].

In this study, we aimed to investigate how resistance can be overcome in chemotherapy‐resistant neuroblastoma cell models. We found two ERBB family members, ERBB4 and ERBB3, and P‐gp to be upregulated in resistant neuroblastoma cell lines. Consistently, co‐treatment of VCR‐resistant neuroblastoma cells with VCR and different ERBB family members or P‐gp inhibitors results in effective breaking of resistance. Functional and knockdown experiments demonstrate P‐gp to be the modulator of this resistance, with ERBB inhibitors showing off‐target or ‘bystander’ P‐gp inhibition. We further show that the cells die by apoptosis once resistance is overcome.

## 2 Materials and methods

### 2.1 Cell lines and cell culture

The VCR‐resistant cell lines BE(2)‐C rVCR, NB‐S‐124 rVCR, NGP rVCR, and MHH‐NB‐11 rVCR were established by continuous exposure to stepwise increasing drug concentrations as previously described [30] and derived from the Resistant Cancer Cell Line (RCCL) Collection [31]. These cell lines and the respective parental cell lines BE(2)‐C control (ATCC, Manassas, VA, USA), NB‐S‐124 control (kindly provided by F. Westermann, DKFZ, Heidelberg, Germany), NGP control (RRID: CVCL_2141; German Collection of Microorganisms and Cell Cultures, DSMZ, Braunschweig, Germany) and MHH‐NB‐11 control (RRID: CVCL_1412; German Collection of Microorganisms and Cell Cultures, DSMZ) were cultured in IMDM medium (#21980‐032, Gibco, Thermo Fisher Scientific Inc., Waltham, MA, USA) supplemented with 10% FCS. To maintain resistance, BE(2)‐C rVCR, NGP rVCR, and MHH‐NB‐11 rVCR were cultured in the presence of 20 ng·mL^−1^ VCR and NB‐S‐124 rVCR in the presence of 100 ng·mL^−1^ VCR.

IMR‐32 (RRID: CVCL_0346) and SK‐N‐BE(2)‐C (RRID: CVCL_0529), both from the German Collection of Microorganisms and Cell Cultures, DSMZ, were kept in DMEM (#41965‐039, Gibco, Thermo Fisher Scientific Inc.) supplemented with 10% FCS and 1% nonessential amino acids (#11140035, Thermo Fisher Scientific Inc.). CHP‐134 (RRID: CVCL_1124), SIMA (RRID: CVCL_1695), and LA‐N‐5 (RRID: CVCL_0389; kindly provided by F. Westermann, DKFZ), as well as SK‐N‐BE(1) (RRID: CVCL_9898), SMS‐KAN (RRID: CVCL_7131), and SMS‐KANR (RRID: CVCL_7132; COG, Philadelphia, PA, USA) and SK‐N‐BE(2) (RRID: CVCL_0528; German Collection of Microorganisms and Cell Cultures, DSMZ), were cultured in RPMI‐1640 (#21875091, Gibco, Thermo Fisher Scientific Inc.) supplemented with 10% FCS. All cells were cultured under standard cell culture conditions. DNA fingerprinting authentication (DSMZ) and bacterial and viral (including mycoplasma) contaminations were regularly checked (Multiplexion, Heidelberg, Germany).

### 2.2 Compounds

Vincristine (VCR, #S2141), afatinib (#S1011), tariquidar (#S8028), lapatinib (#S2111), ceritinib (#S7083), copanlisib (#S2802), and decitabine (#S1200) were purchased from Selleckchem (Houston, TX, USA). Verapamil (#V4629), dactinomycin (#A4262), and hydroxychloroquine (#C6628) were purchased from Sigma–Aldrich (Merck KGaA, Darmstadt, Germany). BKM120 (#11587) and panobinostat (#13280) were purchased from Cayman Chemicals (Ann Arbor, MI, USA). Dasatinib (#D‐3307), everolimus (#E‐4040), and olaparib (#O‐9201) were purchased from LC Laboratories (Woburn, MA, USA). Staurosporine (STS, #HY‐15141) and larotrectinib (#HY‐12866) were purchased from Medchem Express (Monmouth Junction, NJ, USA). Bortezomib (#CT‐BZ001), trametinib (#CT‐GSK112), and venetoclax (#CT‐A199‐2) were purchased from ChemieTek (Indianapolis, IN, USA). The ERBB4 blocking antibody (#MA513016) was purchased from Invitrogen (Thermo Fisher Scientific Inc.).

### 2.3 Metabolic activity assay

Cells were seeded at 1000 cells per well in 25 μL in 384‐well round‐bottom plates and collected by centrifugation (500 × ***g***, 3 min). After 24 h, they were treated using a TECAN D300e digital drug dispenser (Tecan, Männedorf, Switzerland). Metabolic activity was assessed 48 h after treatment using a CellTiter‐Glo® 2.0 kit (Promega, Madison, WI, USA) according to the manufacturer's protocol. Briefly, 15 μL CellTiter‐Glo reagent was added to the plates and mixed by shaking for 5 min at room temperature, followed by a further 15 min incubation at room temperature (light‐protected). Luminescence, corresponding to ATP levels in the cells, was measured using a TECAN Spark plate reader (Tecan) at 28 °C ambient temperature. Metabolic activity (a) and percent inhibition (b) were calculated by the following formulas, where DMSO is the mean of all healthy controls (0.1% DMSO), STS is the mean of all dead controls (1 μm staurosporine) and x are the individual data points:

(1) $$\text{Metabolic activity}=100*\left(1-\left(\left(\text{DMSO}-x\right)/\left(\text{DMSO}-\mathrm{STS}\right)\right)\right)$$

(2) $$\text{Percent inhibition}=100*\left(1-\left(\left(x-\text{DMSO}\right)/\left(\mathrm{STS}-\text{DMSO}\right)\right)\right)$$

### 2.4 Cell viability assay

Cells were seeded in 12‐well plates at 200 000 cells per well and allowed to attach overnight before treatment with technical duplicates. The cells were collected together with their supernatant 48 h after treatment, centrifuged at 350 × ***g*** for 5 min, and re‐suspended in 1 mL medium. Cell viability was assessed with a ViCELL XR automated cell counter (Beckman Coulter Life Sciences, Indianapolis, IN, USA), which counts viable and dead cells differentiated by trypan blue exclusion.

### 2.5 Real‐time RT–PCR

For determination of mRNA levels, 10^6^ cells were collected, and RNA was isolated with the RNeasy Mini Kit (#74106, QIAGEN, Hilden, Germany) according to the manufacturer's instructions, including optional on column DNase digestion (RNase free DNase, #79254, QIAGEN). One microgram of RNA was reverse transcribed to cDNA using the First‐Strand cDNA Synthesis Kit (#K1612, Thermo Fisher Scientific Inc.) following the manufacturer's instructions. For real‐time PCR, 25 ng cDNA and 0.4 μm each of forward and reverse primers were added to the qPCR MasterMix for SYBR Green I (#RT‐SN2x‐O3T, Eurogentec, Seraing, Belgium), and amplification was measured using a 7500 Real Time PCR System by Applied Biosystems (Thermo Fisher Scientific Inc.). Thermocycling conditions were 2 min at 50 °C, 10 min at 95 °C, 40 cycles of 15 s at 95 °C and 1 min at 60 °C, and 30 s at 95 °C and 15 s at 60 °C. The following primers were used: *ABCB1* forward: 5’‐GGGATGGTCAGTGTTGATGGA‐3′; *ABCB1* reverse: 5’‐GCTATCGTGGTGGCAAACAATA‐3′; *ERBB4* forward: 5’‐GCCTGTCCTTGCTTATCCTCAA‐3′; *ERBB4* reverse: 5’‐CCTGCGCTGATTTCCTTCA‐3′; *EGFR* forward: 5’‐GGAGAACTGCCAGAAACTGACC‐3′; *EGFR* reverse: 5’‐GCCTGCAGCACACTGGTTG‐3′; *ERBB2* forward: 5’‐GGGAAGAATGGGGTCGTCAAA‐3′; *ERBB2* reverse: 5’‐CTCCTCCCTGGGGTGTCAAGT‐3′; *ERBB3* forward: 5’‐GGTGATGGGGAACCTTGAGAT‐3′; *ERBB3* reverse: 5’‐CTGTCACTTCTCGAATCCACTG‐3′; *HPRT* forward: 5’‐TGACACTGGCAAAACAATGCA‐3′; *HPRT* reverse: 5’‐GGTCCTTTTCACCAGCAAGCT‐3′; *SDHA* forward: 5’‐TGGGAACAAGAGGGCTGCTG‐3′; *SDHA* reverse: 5’‐CCACCACTGCATCAAATTCATG‐3′. *SDHA* and *HPRT* were used as housekeeping genes [32], and fold changes were calculated based on the 2^−ΔΔCT^ method [33]. Unless stated otherwise, a mixture of cDNA from untreated LAN‐5, NB‐1, IMR‐32, Kelly, and SK‐N‐BE(2)‐C cells was used as an internal reference to which all values were normalized.

### 2.6 ERBB4 and ABCB1 knockdown

For the knockdown followed by viability assay, 60 000 cells per well were seeded in 6‐well plates. For the knockdown followed by colony assay, 800 cells per well were seeded in 6‐well plates. For the knockdown followed by calcein efflux assay or for the knockdown control by western blot, 200 000 cells per plate were seeded in 10‐cm dishes. Cells were allowed to attach for 24 h and transfected with 25 nm siRNA in OptiMEM (#31985‐047, Gibco, Thermo Fisher Scientific Inc.) with 0.5% HiPerFect (#301704, QIAGEN) overnight, followed by the respective treatments or medium change for western blot control. The following siRNAs were used: ERBB4 siRNAs #4 (SI00074214), #5 (SI02223893), and #10 (SI04435067) from QIAGEN were pooled for *ERBB4* knockdown, smart pool ABCB1 siRNA (L‐003868‐00‐0005) from Dharmacon (Lafayette, CO, USA) for *ABCB1* knockdown, and negative control siRNAs (#1 and #5 from Ambion, Austin, TX, USA) were pooled for control transfection.

### 2.7 Western blotting

For western blotting, 10^6^ cells were seeded in 10 cm dishes and treated after 24 h. For cell death analysis, cells were harvested, including the supernatant, and collected at 350 × ***g*** for 5 min at 4 °C before lysis. For ERBB4 and P‐gp levels and for cell death analysis, cells were lysed in SDS buffer (62.5 mm Tris, pH 6.8, 2% SDS, 10% glycerol, 0.1 m DTT), followed by 5 min at 95 °C. Debris was pelleted by centrifugation at 10 000 × ***g*** for 10 min at 10 °C, and the supernatant was collected.

For analysis including phosphoproteins, cells were lysed on ice for 20 min in NP‐40 buffer (50 mm Tris, pH 8, 150 mm NaCl, 1% NP‐40) with phosphatase inhibitors (PhosSTOP EASYPack, #04906845001, Roche Diagnostics GmbH, Mannheim, Germany) and protease inhibitors (cOmplete protease inhibitor cocktail, #11697498001, Roche Diagnostics GmbH). Debris was pelleted by centrifugation at 20 000 × ***g*** for 15 min at 4 °C, and the supernatant was collected. Before SDS PAGE, 4× Laemmli (62.5 mm Tris, pH 6.8, 20% glycerol, 4% SDS, 5% beta‐mercaptoethanol, bromophenol blue) was added to the sample, followed by incubation at 95 °C for 5 min.

The following primary antibodies were used (diluted 1 : 1000 unless otherwise indicated): mouse anti‐GAPDH (diluted 1 : 40 000, #MAB374, Merck KGaA), mouse anti‐PARP (#556494, BD Biosciences, Heidelberg, Germany), rabbit anti‐p‐SRC (Y419; diluted 1 : 200, #AF2685, R&D Systems, Minneapolis, MN, USA), and mouse anti‐transferrin receptor (#13‐6800, ThermoFisher Scientific Inc.). Rabbit anti‐AKT (#9272), rabbit anti‐p‐AKT (S473) (#9271), rabbit anti‐BID (#2002), rabbit anti‐ERBB4 (#4795), rabbit anti‐ERK (#4695), rabbit anti‐p‐ERK (T202/Y204) (#4370), rabbit anti‐SRC (#2123), and rabbit P‐glycoprotein (#13978s) were purchased from Cell Signaling Technology (Danvers, MA, USA). Secondary antibodies were diluted 1:60 000: donkey anti‐rabbit IgG HRP (#31.458, ThermoFisher Scientific Inc.) and goat anti‐mouse IgG HRP (#115‐035‐003, DIANOVA, Hamburg, Germany). The Precision Plus Protein™ Kaleidoscope™ Prestained Protein Standard (#1610375, Bio‐Rad, Munich, Germany) was loaded to monitor protein separation and estimate the molecular weight of sample proteins. The complete images of all western blots shown are included in the supplemental material. Image quantification was performed in imagej (version 1.53e; National Institutes of Health, Bethesda, MD, USA).

### 2.8 Immunofluorescence microscopy

For immunofluorescence microscopy, 2500 cells per well were seeded in a flat‐bottom 384‐well plate and allowed to attach overnight. Cells were then fixed in 4% PFA, permeabilized in 0.1% Triton/PBS and blocked in 3% BSA + 0.05% Triton in PBS. The primary antibody rabbit anti‐ERBB4 (#4795, Cell Signaling Technology) was diluted 1 : 100, and the primary antibody mouse anti‐P‐glycoprotein (#Ab00143‐1.1, Absolut Antibody Ltd, Redcar, UK) was diluted 1 : 250. Secondary antibodies donkey anti‐rabbit IgG labeled with Alexa Fluor 508 (#A10042, ThermoFisher Scientific Inc.) and goat anti‐mouse IgG labeled with Alexa Fluor 488 (#4408S, Molecular Probes, ThermoFisher Scientific Inc.) were diluted 1 : 500 and 1 : 1000, respectively, and stained concurrently with 0.5 μg·mL^−1^ Hoechst 33342 (#H3570, ThermoFisher Scientific Inc.). Between steps, the cells were washed three times with PBS. Images were taken using an ImageXpress Micro Confocal high content microscope (Molecular Devices, San Jose, CA, USA) using Cy3 (ERBB4), FITC (P‐gp), DAPI (Hoechst 33342), and brightfield channels.

### 2.9 P‐gp surface expression

For measurement of surface P‐gp, 2 × 10^6^ cells were seeded in 10 cm dishes. Cells were collected in phenol red‐free RPMI and, except the controls, incubated with mouse P‐glycoprotein antibody (#Ab00143‐1.1, Absolut Antibody Ltd) for 2 h on ice, followed by washing steps and APC‐labeled goat anti‐mouse IgG secondary antibody (#115‐136‐068, DIANOVA) for 2 h on ice. Cells were washed, and surface P‐gp was measured with excitation at 640 nm and 670/14 nm bandpass filters on a FACSCanto II (BD Biosciences).

### 2.10 P‐gp function (calcein efflux assay)

To assess P‐gp function, 500 000 cells per well were seeded in 6‐well plates and allowed to attach overnight before treatment for 24 h. The cells were then stained with 10 nm of the P‐gp substrate calcein AM (#65‐0853‐39, Thermo Fisher Scientific Inc.) in phenol red‐free RPMI (#11835030, Gibco, Thermo Fisher Scientific Inc.) with 10% FCS for 15 min at 37 °C. To assess P‐gp function after *ABCB1* knockdown, cells were stained with 100 nm calcein for 30 min at 37 °C. The cells were washed, and the remaining intracellular calcein was measured with excitation at 488 and 502 nm longpass and 530/30 nm bandpass filters on a FACSCanto II (BD Biosciences).

### 2.11 Colony assay

For the colony assay, 800 cells per well were seeded in 6‐well plates and allowed to attach for 24 h before *ABCB1* knockdown. Twenty‐four hours after knockdown, the cells were treated for 72 h, followed by medium change and colony growth for 9 days. Colonies were stained with 1% crystal violet in 70% ethanol, washed with dH_2_O, dried, and scanned. The colony number was evaluated with imagej (version 1.53e; National Institutes of Health).

### 2.12 Caspase activity assay

For the caspase activity assay, 10^6^ cells were seeded in 10 cm dishes and allowed to attach overnight before treatment for 48 h in technical duplicates. Protein lysates of all adherent and detached cells were taken in cell lysis buffer (#BV‐1067‐100, Axxora (Enzo Life Sciences GmbH, Lörrach, Germany)). Ten millimolar DTT and 50 μm caspase‐3/7 fluorogenic substrate (Ac‐DEVD‐AFC, #556574, BD Pharmingen™, BD Biosciences) were added to 10 μg of protein in reaction buffer (#JM‐1068‐20, MBL International, Woburn, MA, USA). Fluorescent readings were taken in technical duplicates at a FLUOstar OPTIMA plate reader (BMG LABTECH GmbH, Ortenberg, Germany) at 37 °C with excitation of 380 nm and emission at 520 nm in 10 min intervals for 3 h until the signal saturated.

### 2.13 BODIPY lipid peroxidation measurement

For lipid peroxidation measurement, 500 000 cells per well were seeded in 6‐well plates and allowed to attach for 24 h, followed by 48 h of treatment. Cells were collected with their supernatant and stained with 20 μm BODIPY 581/591 Lipid Peroxidation Sensor stain (#D3861, ThermoFisher Scientific Inc.) in phenol red‐free RPMI medium (#11835030, Gibco, ThermoFisher Scientific Inc.) with 10% FCS for 30 min at 37 °C. Cells were washed, and oxidized BODIPY was measured with excitation at 488 and 502 nm longpass and 530/30 nm bandpass filters at a FACSCanto II (BD Biosciences).

### 2.14 Zebrafish embryo xenograft model

Wild‐type zebrafish embryos of the AB line (European Zebrafish Resource Center, EZRC, Eggenstein‐Leopoldshafen, Germany) were used for xenograft experiments. The fish were cared for and bred under standard conditions, as described previously [34]. Briefly, fish were raised and bred at 28 °C, and for the experiments, eggs were collected and placed in E3 embryonic buffer. Twenty‐four hours post‐fertilization (hpf), 0.2 mm 1‐phenyl‐2‐thiourea (PTU, Sigma–Aldrich, Merck KGaA) was added.

The toxicity of VCR and tariquidar to the embryos was tested before the start of the xenograft experiments. For this, embryos were exposed to the compounds at 48 hpf and imaged with a stereomicroscope (Leica) at 72 and 120 hpf. Assessment of toxicity included death, morphological changes (e.g., edema or curvature of body), and behavioral changes. None of the fish embryos showed toxicity at any of the concentrations used.

The xenograft experiments were performed as described previously [34]. In short, cells were harvested at 70–80% confluency and fluorescently labeled with CellTracker CM‐DiI (#C7000, Thermo Fisher Scientific Inc.). To avoid cell clumps, cells were incubated with DNase I and, after washing steps, resuspended at 10^8^ cells·mL^−1^ in serum‐free RPMI. Zebrafish embryos were anesthetized with tricaine at 48 hpf and subsequently embedded in 1% low gelling temperature agarose (Sigma–Aldrich, Merck KGaA). A total of 150–250 fluorescently labeled cells were injected into the yolk sack of each zebrafish embryo with microinjection needles (Science Products, Hofheim, Germany) and a FemtoJet express microinjector (Eppendorf, Hamburg, Germany). After injection, the embryos were kept at 34°C. Initial tumor volume was assessed with a Zeiss LSM 710 confocal microscope (Zeiss, Oberkochen, Germany) 24 h post‐injection (hpi), after which they were exposed to treatment. The treatment effect on tumor volume was quantified after 48 h (120 hpf). Tumor development was rated for each embryo individually by calculating the change from baseline according to the following formula, where Volume Day 1 and Volume Day 3 are the tumor volumes 24 and 72 h after injection, respectively:

(3) $$\text{Change from baseline}=100\%*\left(\text{Volume}\;\mathrm{Day}1/\text{Volume}\;\mathrm{Day}3\right)–100\%$$

### 2.15 Analysis of neuroblastoma gene expression data from R2

#### 2.15.1 Cell lines

Data were retrieved from ‘R2: Genomics Analysis and Visualization Platform’ (http://r2.amc.nl). The specific cell line datasets used were ‘Cell line Neuroblastoma ‐ Utnes ‐ 10 ‐ deseq2 ‐ ravi002’ (GEO ID: GSE148700 [35], accessed 07 January 2021), ‘Cell line Neuroblastoma ‐ Jagannathan ‐ 38 ‐ custom ‐ ilmnhwg6v2’ (GEO ID: GSE19274 [36], accessed 08 June 2021), ‘Cell line Neuroblastoma ‐ Maris ‐ 41 ‐ FPKM ‐ rsg001’ (GEO ID: GSE89413 [37], accessed 07 January 2021), ‘Cell line Neuroblastoma ‐ Versteeg ‐ 24 ‐ MAS5.0 ‐ u133p2’ (GEO ID: GSE28019, accessed 07 January 2021) and ‘Cell line CCLE Cancer Cell Line Encyclopedia ‐ Broad ‐ 917 ‐ MAS5.0 ‐ u133p2’ (GEO ID: GSE36133 [38], accessed 08 June 2021). Only cell lines that could be positively identified as having been generated before (primary) or after (relapse) the patient had received chemotherapy were included.

#### 2.15.2 Patient samples

The ‘Tumor Neuroblastoma Prim Relapse ‐ Schramm ‐ 18 ‐ custom ‐ ag44kcwolf’ patient dataset (GEO ID: GSE65303 [39], accessed 09 September 2021) and unpublished data from the INFORM personalized medicine study [40, 41] were used.

For the analysis, all datasets were reduced to the members of the ERBB family and the ABC family. Group means of primary and relapsed cells or samples were calculated and tested for differences using ANOVA followed by Tukey's honestly significant difference post‐test using r (version 4.0.3). As the INFORM dataset includes mostly relapsed tumors, expression was compared between neuroblastoma and other pediatric tumor entities.

### 2.16 Institutional review board statement

Zebrafish husbandry (permit number 35‐9185.64/BH Hassel/Meder) and experiments (permit number 35‐9185.81/G‐126/15) were performed according to local animal welfare standards (Tierschutzgesetz §11, Abs. 1, No. 1) and in accordance with European Union animal welfare guidelines (EU Directive 2010/63/EU). All applicable national and institutional guidelines for the care and use of zebrafish were followed. All procedures involving animals were performed in accordance with the ethical standards of the Medical Faculty University Hospital Heidelberg.

### 2.17 Written informed consent statement and ethical approval

The study was conducted in accordance with Good Clinical Practice guidelines and the Declaration of Helsinki. All patients or their legally acceptable representative, or both (if possible), provided written informed consent. Approvals for the study protocol (and any modifications thereof) were obtained from independent ethics committees and the institutional review board at each participating center. The INFORM Registry was registered with the German Clinical Trial Register, number DRKS00007623.

### 2.18 Statistics

Synergy scores were calculated based on the Bliss reference model using synergyfinder [42]. All other statistics were calculated in r (version 4.0.3). ANOVA with Tukey's post‐test and *t* test were calculated using the core ‘stats’ package. Curve fit and IC50 calculations were performed with the ‘drc’ package (version 3.0–1) using the four‐parameter log‐logistic function. AUCs were calculated using the ‘pharmacogx’ package (version 2.2.4). If necessary, outlier calculations were performed using the Grubbs test in the ‘outliers’ package (version 0.14). Figures were generated using the ‘ggplot2’ package (version 3.3.5).

## 3 Results

### 3.1 Chemotherapy resistance is broken by inhibitors of the ERBB family and P‐gp

**Fig. 1:**
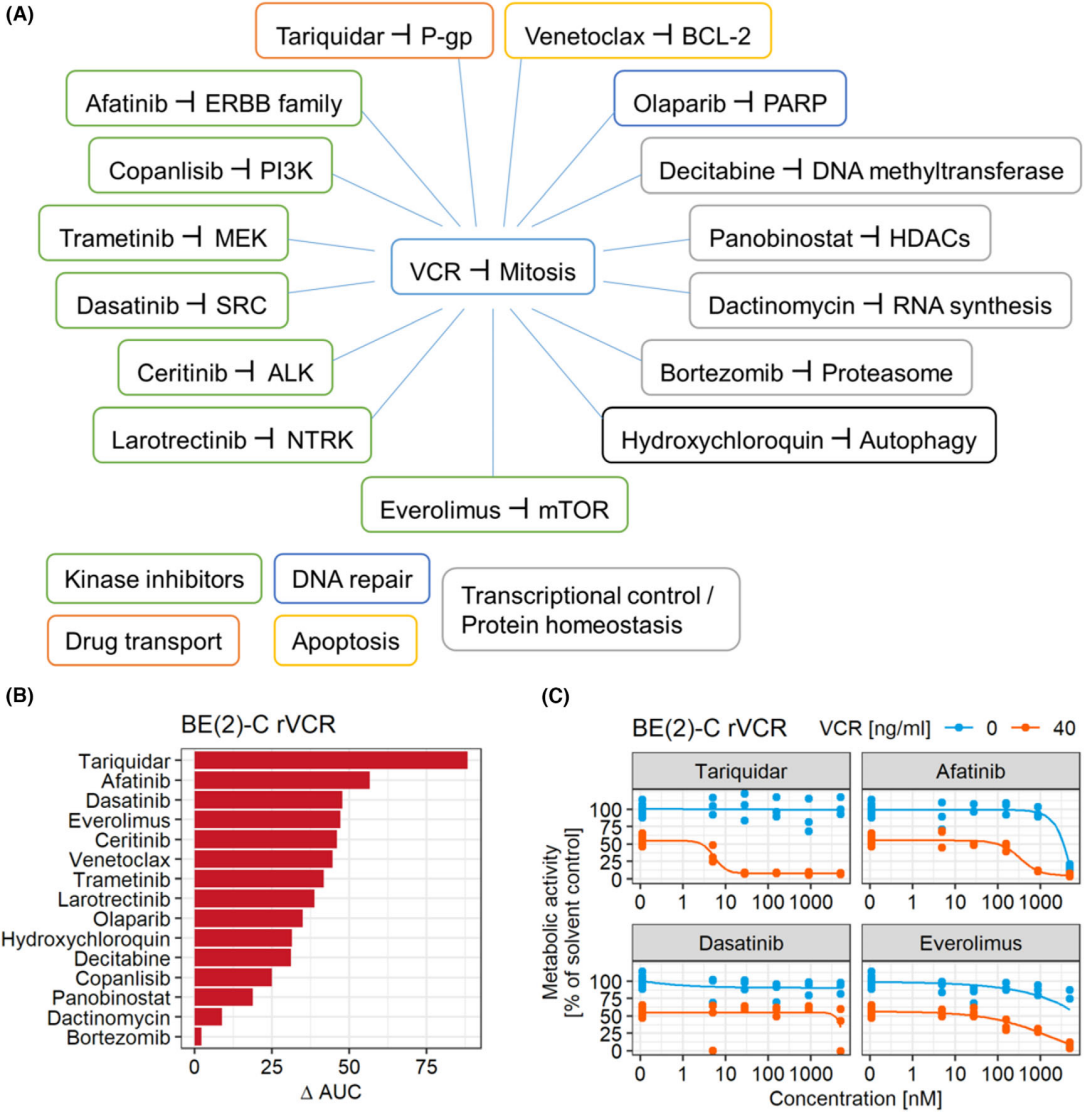
Functional screen identifies ERBB4 and P‐gp inhibitors to break resistance. (A) Schematic representation of the drugs and their targets. Drug classes are indicated. (B) A metabolic activity screen read‐out (CellTiterGlo) of combination treatment of VCR with 15 anticancer drugs (14 clinically approved) was performed with BE(2)‐C rVCR. The cells were treated with 0, 5, 28, 158, 889, and 5000 nm of the 15 drugs alone or in combination with 40 ng·mL^−1^ VCR for 48 h. The difference in area under the curve (AUC) between single and combination treatment (ΔAUC) was calculated. The screen was performed in triplicates. (C) Concentration curves of the top four AUC hits (in triplicates) from (B) with (orange) or without (blue) addition of 40 ng·mL^−1^ VCR.

To investigate how chemotherapy resistance may be overcome in high‐risk neuroblastoma, we studied a vincristine (VCR)‐resistant subline of the well‐established high‐risk neuroblastoma cell line SK‐N‐BE(2)‐C. We performed a metabolic activity screen with 14 clinically approved drugs targeting a wide range of cancer‐ and neuroblastoma‐relevant pathways, such as ALK, MAPK, and PI3K, to identify agents that can overcome VCR resistance (Fig. 1A). Since VCR resistance is known to be mediated by the transmembrane pump P‐gp, we additionally included the P‐gp inhibitor tariquidar (XR9576), which has been studied in clinical trials (e.g., NCT00011414). We treated BE(2)‐C rVCR with five concentrations of each compound alone or in combination with 40 ng·mL^−1^ VCR and calculated the respective areas under the curve (AUCs). The greatest difference between the AUCs of the single compound and combination treatment (ΔAUC) was found for tariquidar, followed by afatinib (Fig. 1B). Afatinib is a potent inhibitor of the ERBB family, with a particularly high affinity for EGFR and ERBB4 [43]. The metabolic activity curves for single and combination treatments of the four compounds with the highest ΔAUC are exemplarily shown in Fig. 1C. The low ΔAUC for bortezomib stems from the strong effect of bortezomib itself, which was not further enhanced by the addition of VCR. Because afatinib is a clinically approved drug combined with a high ΔAUC, we pursued the ERBB family as a potential mechanism of resistance.

**Fig. 2:**
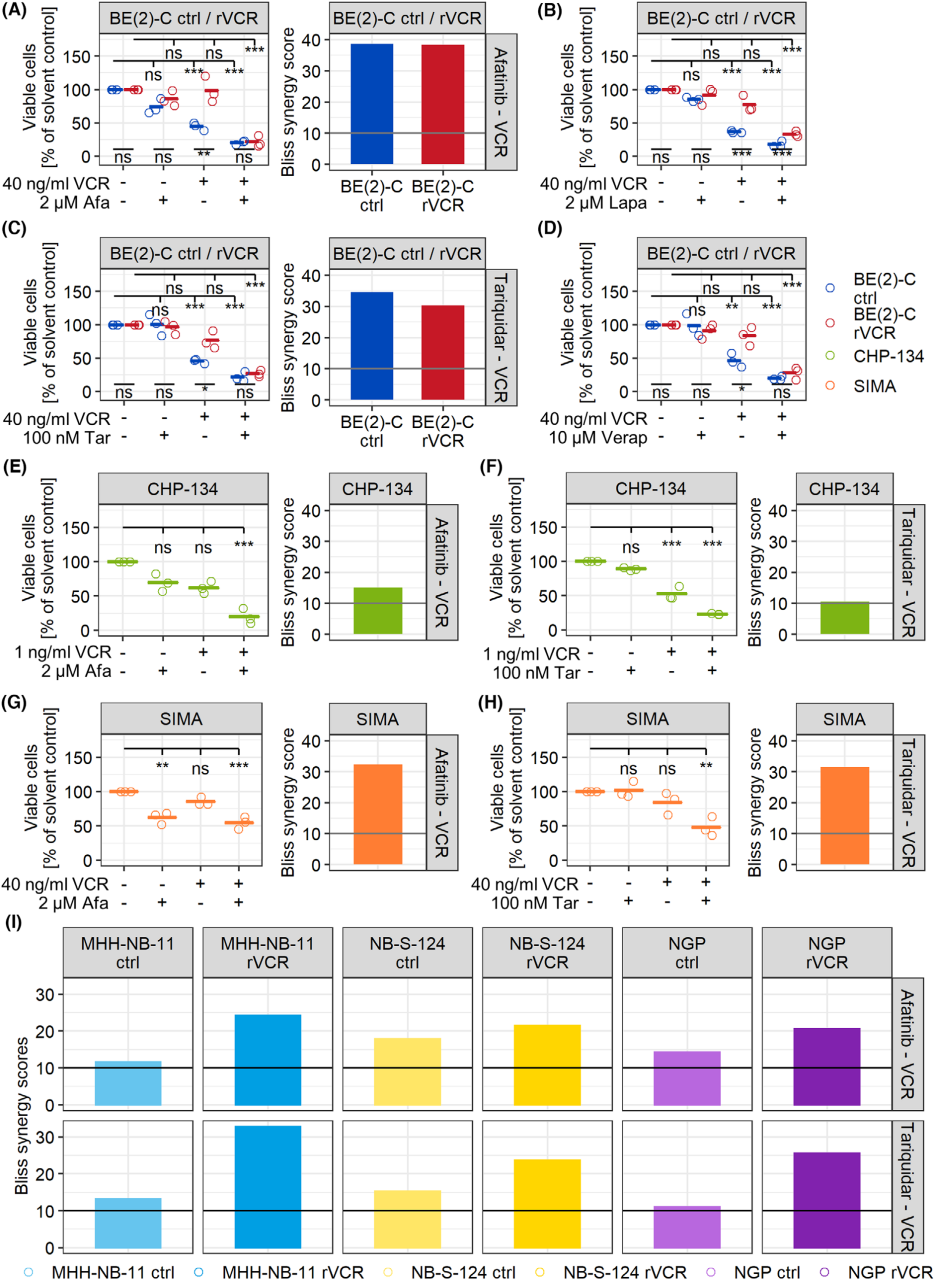
Inhibitors of the ERBB family and P‐gp break resistance in BE(2)‐C rVCR. (A–D) BE(2)‐C control and rVCR cells were treated with 40 ng·mL^−1^ VCR and the indicated concentrations of the ERBB inhibitors afatinib (A) and lapatinib (B) or the P‐gp inhibitors tariquidar (C) or verapamil (D) for 48 h. (E, F) CHP‐134 cells were treated with 1 ng·mL^−1^ VCR and 2 μm afatinib (E) or 100 nm tariquidar (F) for 48 h. (G, H) SIMA cells were treated with 40 ng·mL^−1^ VCR and 2 μm afatinib (G) or 100 nm tariquidar (H) for 48 h. (A–H) The number of viable cells was determined by trypan blue exclusion. The percent of viable cells relative to the solvent control of each cell line was calculated. Presented are biological replicates (*n* = 3) and their means. Statistics were calculated with ANOVA followed by Tukey's post‐test. Shown are the statistics for the comparisons between the cell lines (bottom) as well as the comparisons to solvent control for each cell line (top). (A–I) Bliss synergy scores for the indicated cell lines were calculated from metabolic activity data (0, 10, 20, 40, 100 ng·mL^−1^ VCR ±0, 0.5, 1, 2 μm afatinib or 0, 1, 10, 1000 nm tariquidar for 48 h in all cell lines) using synergyfinder. A score > 10 (marked with a line) indicates synergy. ***P < 0.001, **P < 0.01, *P < 0.05, ns, not significant.

To validate these findings, viable cell counts by trypan blue exclusion were performed. As expected, BE(2)‐C rVCR (red circles) were resistant to 40 ng·mL^−1^ VCR compared to the sensitive BE(2)‐C ctrl (blue circles; Fig. 2A–D), while both cell lines responded similarly to afatinib (Fig. 2A). The combination of VCR with afatinib reduced the viability of BE(2)‐C rVCR to the level of the control cell line (Fig. 2A), hence efficiently breaking the existing VCR resistance. The same effect was observed with lapatinib, another pan‐ERBB family inhibitor with a high affinity for EGFR and ERBB4 (Fig. 2B). Synergy was validated with a metabolic activity readout using the Bliss synergy model (Figs 2A and S1A). Bliss synergy scores above 10 indicate synergy, and scores below −10 indicate antagonism. High synergy scores above 30 indicated strong synergy between afatinib and VCR in both models (Fig. 2A and Table 1).

**Table 1:** The combination of VCR with afatinib or tariquidar is synergistic in neuroblastoma cell lines. Ctrl, control; rVCR, vincristine resistant.

Cell line	Drug combination	Bliss synergy score^a^
BE(2)‐C ctrl	Afatinib ‐ VCR	38.65
BE(2)‐C ctrl	Tariquidar ‐ VCR	34.59
BE(2)‐C rVCR	Afatinib ‐ VCR	38.43
BE(2)‐C rVCR	Tariquidar ‐ VCR	30.39
CHP‐134	Afatinib ‐ VCR	15.13
CHP‐134	Tariquidar ‐ VCR	10.58
MHH‐NB‐11 ctrl	Afatinib ‐ VCR	11.60
MHH‐NB‐11 ctrl	Tariquidar ‐ VCR	13.20
MHH‐NB‐11 rVCR	Afatinib ‐ VCR	24.21
MHH‐NB‐11 rVCR	Tariquidar ‐ VCR	32.83
NB‐S‐124 ctrl	Afatinib ‐ VCR	17.87
NB‐S‐124 ctrl	Tariquidar ‐ VCR	15.34
NB‐S‐124 rVCR	Afatinib ‐ VCR	21.50
NB‐S‐124 rVCR	Tariquidar ‐ VCR	23.69
NGP ctrl	Afatinib ‐ VCR	14.30
NGP ctrl	Tariquidar ‐ VCR	11.00
NGP rVCR	Afatinib ‐ VCR	20.56
NGP rVCR	Tariquidar ‐ VCR	25.57
SIMA	Afatinib ‐ VCR	32.27
SIMA	Tariquidar ‐ VCR	31.51

a Synergy scores were calculated using synergyfinder. A synergy score > 10 indicates synergism, a synergy score between −10 and 10 indicates additivity, and a synergy score < −10 indicates antagonism.

As the P‐gp inhibitor tariquidar had the highest ΔAUC, we also examined P‐gp as a resistance mechanism. We, therefore, treated the cells with a combination of VCR and the P‐gp inhibitors tariquidar or verapamil. P‐gp inhibition alone did not affect cell viability but, similar to afatinib and lapatinib, was able to efficiently overcome resistance in BE(2)‐C rVCR when combined with VCR (Fig. 2C,D), corroborated by strong synergism for the combination of VCR with tariquidar (Figs 2C and S1A, and Table 1).

To exclude a single‐cell line effect, we performed further trypan blue exclusion assays in the neuroblastoma cell lines CHP‐134 and SIMA, where we observed a similar decrease in viability when treated with VCR in combination with afatinib or tariquidar (Fig. 2E–H). We confirmed synergism by metabolic activity assays and the Bliss model (Figs 2E–H and S1A, and Table 1). Three other sensitive/resistant neuroblastoma cell line pairs (MHH‐NB‐11 ctrl & rVCR, NB‐S‐124 ctrl & rVCR and NGP ctrl & rVCR) also showed synergism between VCR and afatinib or tariquidar (Figs 2I and S1B, and Table 1).

### 3.2 ERBB3, ERBB4, and P‐gp are upregulated in therapy‐resistant neuroblastoma cell lines

**Fig. 3:**
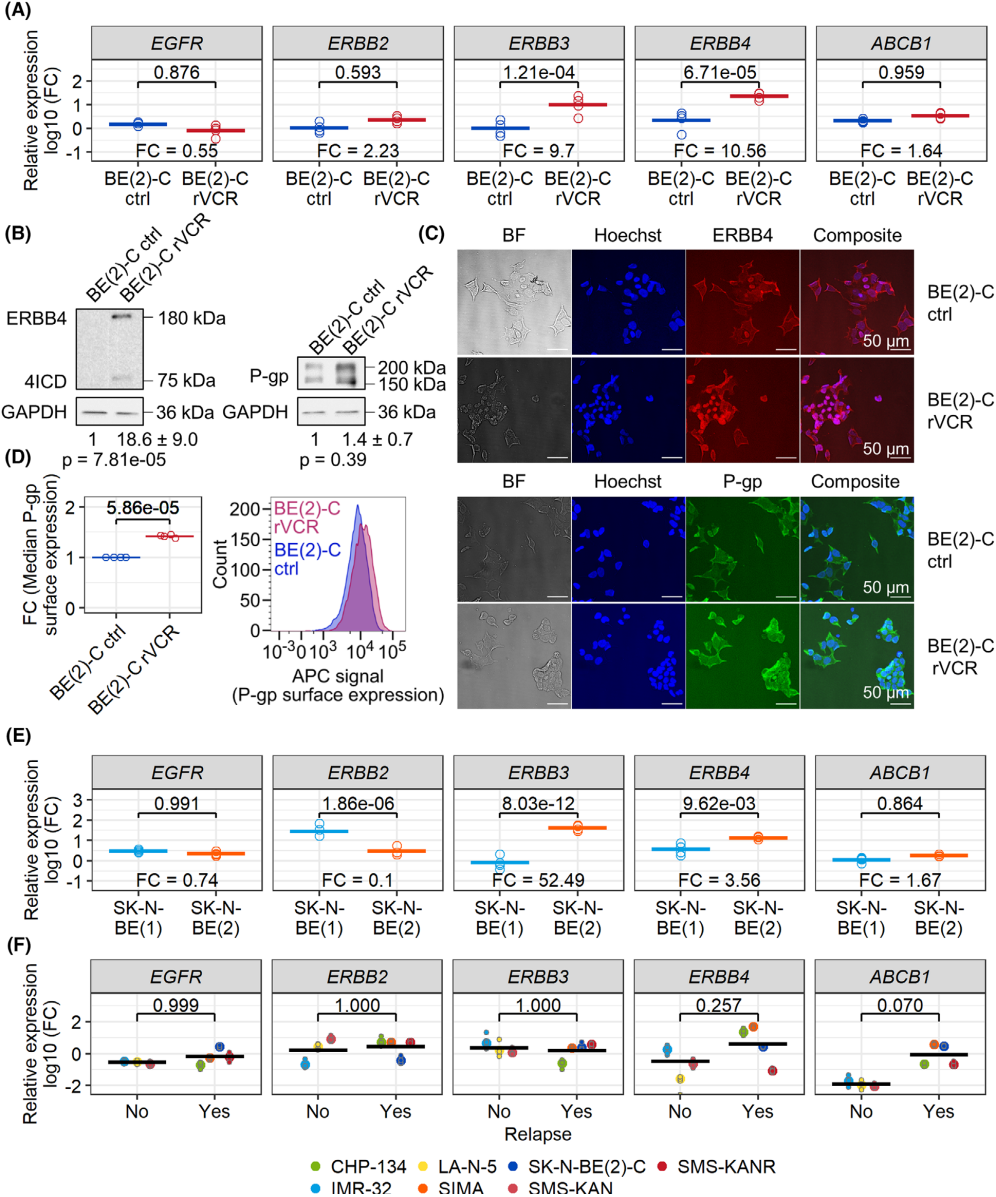
ERBB3, ERBB4, and P‐gp/*ABCB1* are upregulated in resistant BE(2)‐C and SK‐N‐BE(2). (A) Real‐time PCR of the indicated genes was performed on cDNA of untreated control BE(2)‐C and BE(2)‐C rVCR. Expression is normalized to a mix of five neuroblastoma cell lines. *P* values are indicated and were calculated with ANOVA followed by Tukey's post‐test. The fold change between control and resistant BE(2)‐C is likewise indicated. Biological replicates (*n* = 4) and their means are shown. (B) Western blotting against ERBB4 and P‐gp was performed with untreated control BE(2)‐C and BE(2)‐C rVCR. Depicted are representative blots of at least four biological replicates. Quantifications were normalized first to the respective GAPDH and then to control BE(2)‐C. Noted are the means of the replicates and their standard deviations. Significance was calculated by Student's *t* test against 0 of the log10 transformed fold change values of BE(2)‐C rVCR. (C) Representative immunofluorescence images of untreated control BE(2)‐C and BE(2)‐C rVCR (12 technical replicates each). Cells were labeled with ERBB4 or P‐gp primary antibody and stained with Alexa Fluor 508 (ERBB4)‐ or Alexa Fluor 488 (P‐gp)‐labeled secondary antibodies, as well as the DNA intercalating dye Hoechst 33342. The white scale bar indicates 50 μm. (D) BE(2)‐C ctrl and rVCR were immunostained with P‐gp primary antibody and APC‐labeled secondary antibody, and surface P‐gp was analyzed with flow cytometry. The right panel shows a representative signal distribution of *n* = 4 independent replicates. The median APC signal normalized to control BE(2)‐C is depicted in the left panel. The four replicates and their means are shown. Significance was calculated by Student's *t* test against 0 of the log10 transformed fold change values of BE(2)‐C rVCR. (E) Real‐time PCR of the indicated genes was performed on untreated SK‐N‐BE(1) and SK‐N‐BE(2) cells. Expression is normalized to a mix of five neuroblastoma cell lines. *P* values are indicated and were calculated by ANOVA followed by Tukey's post‐test. The fold change between SK‐N‐BE(1) and SK‐N‐BE(2) is likewise indicated. Biological replicates (*n* = 4) and their means are shown. (F) Real‐time PCR of the indicated genes was performed on a panel of three non‐relapsed and four relapsed neuroblastoma cell lines. Expression is normalized to a mix of five neuroblastoma cell lines. The biological replicates of each cell line (*n* ≥ 3), the mean relative expression of each cell line, and the group means are shown. *P* values between group means are indicated and were calculated by ANOVA followed by Tukey's post‐test.

Having determined that both the ERRB family inhibitors afatinib and lapatinib and the P‐gp inhibitors tariquidar and verapamil are very efficient in breaking VCR resistance, we wanted to identify the role of the individual members of the ERBB family and P‐gp (*ABCB1*). We therefore performed real‐time PCR experiments to examine ERBB family member mRNA levels in control BE(2)‐C and BE(2)‐C rVCR cells. *ERBB4* and *ERBB3* were significantly upregulated in BE(2)‐C rVCR cells compared to control cells, while *EGFR*, *ERBB2*, and *ABCB1* were not differentially expressed between the cell lines at the mRNA level (Fig. 3A). Western blots and immunofluorescence staining confirmed that the afatinib‐target ERBB4 was highly expressed in BE(2)‐C rVCR (Fig. 3B,C). Western blot of complete cell lysates showed P‐gp levels to be slightly but not significantly increased in BE(2)‐C rVCR (Fig. 3B), while immunofluorescence microscopy of cell surface P‐gp as well as flow cytometric analysis confirmed a significant increase in surface P‐gp in BE(2)‐C rVCR (Fig. 3C,D).

SK‐N‐BE(2)‐C cells are a subclone of the cell line SK‐N‐BE(2), which was derived after treatment from the same patient from whom the cell line SK‐N‐BE(1) had been derived at primary diagnosis. We therefore examined whether this primary‐relapse pair showed a similar expression pattern for the ERBB family and *ABCB1*. Indeed, the SK‐N‐BE(1)/SK‐N‐BE(2) pair displayed a similar mRNA expression profile as the BE(2)‐C control/BE(2)‐C rVCR pair: *ERBB3* and *ERBB4* were significantly upregulated in SK‐N‐BE(2) compared to SK‐N‐BE(1), while *EGFR*, *ERBB2*, and *ABCB1* were not (Fig. 3E).

As all four cell lines were derived from the same patient, we tested a panel of seven further cell lines, three derived at primary diagnosis and four derived at relapse. Real‐time PCR analyses of these cell lines showed increased expression of *ERBB4* and *ABCB1*, although this effect did not reach statistical significance (Fig. 3F).

In summary, we found ERBB4 to be upregulated at the mRNA level and at the protein level in both the VCR‐resistant BE(2)‐C rVCR and in the relapsed cell line SK‐N‐BE(2) compared to the control BE(2)‐C and SK‐N‐BE(1), respectively. While *ABCB1* was not upregulated at the mRNA level, the surface expression of P‐gp was increased in BE(2)‐C rVCR.

### 3.3 ABCB1 is upregulated in relapsed neuroblastoma

To systematically investigate the expression of the ERBB family and *ABCB1* in a larger number of samples, we analyzed several gene expression datasets from R2.

We independently analyzed several cell line datasets provided by Jagannathan et al. (GSE19274) [36], Versteeg et al. (GSE28019), Maris et al. (GSE89413) [37], and the Cell Line Encyclopedia from the Broad Institute (GSE36133) [38]. In all cases, only those neuroblastoma cell lines were included in the analysis that could be identified as having been derived before (primary) or after (relapse) the patient had received chemotherapy. All four datasets confirmed no difference in the expression of any ERBB family member between the two groups, while *ABCB1* was upregulated at relapse (Fig. S2A–D). In the two datasets containing SK‐N‐BE(2)‐C (Jagannathan and Maris), this cell line was among the four cell lines with the highest *ABCB1* expression.

The dataset provided by Utnes et al. is unique, as it directly compares five cell line pairs derived at primary and relapse from the same patients (GSE148700) [35]. Again, the group means for the genes of the ERBB family were not different. However, the SK‐N‐BE(1)/SK‐N‐BE(2) pair showed a clear upregulation of *ERBB4* (Fig. S2E), confirming our real‐time PCR results. *ABCB1* levels were either upregulated or stable at relapse in each cell line pair.

**Fig. 4:**
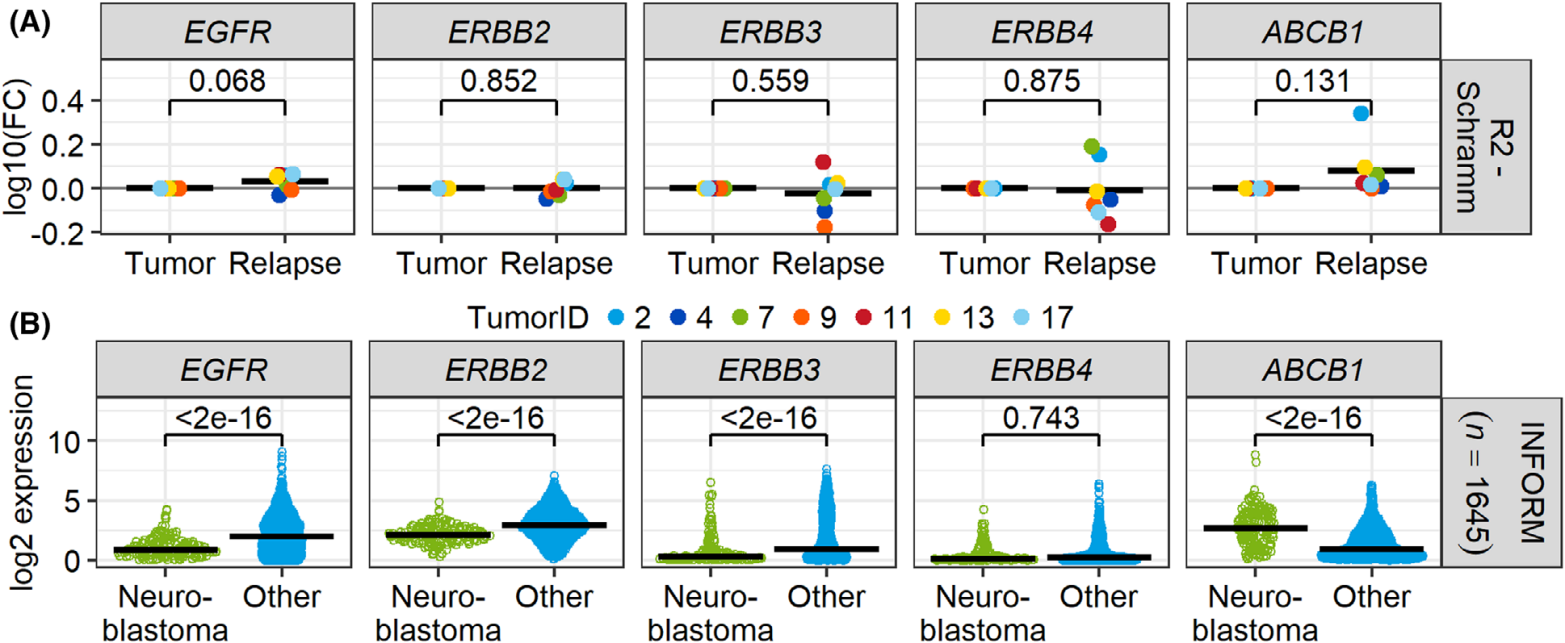
ABCB1 is upregulated in neuroblastoma relapsed tumors. (A) Gene expression data of 18 tumor samples from the dataset by Schramm et al. (GSE65303) were downloaded from R2 (http://r2.amc.nl, 09 September 2021). Only paired samples with data at primary diagnosis (tumor) and at relapse were included (*n* = 7 pairs). Statistics of log‐transformed relapse values were calculated with Student's *t* test against 0. (B) Gene expression data from the INFORM registry (*n* = 1645). Comparison of neuroblastoma cases (*n* = 162) vs. all other entities. Statistics were calculated by ANOVA followed by Tukey's post‐test including all genes of the ERBB and ABC families present in the respective datasets.

Moreover, we analyzed gene expression data obtained from patient samples. The dataset collected by Schramm et al. (GSE65303) [39] contains data from seven neuroblastoma patients before and after chemotherapy. In this cohort, one patient displayed strong *ERBB3* and two patients displayed strong *ERBB4* upregulation in the relapsed tumor compared to the primary tumor. However, no member of the ERBB family was coherently regulated between the groups when comparing the means. *ABCB1* was upregulated or not altered in the relapsed tumors compared to their primary tumors. Comparing the means, *ABCB1* was upregulated (*P* = 0.131; Fig. 4A). Notably, using expression data from the Individualized Therapy For Relapsed Malignancies in Childhood (INFORM) register (*n* = 1483) [40, 41], the expression of *ABCB1* expression was significantly higher (*P* < 0.001) in relapsed neuroblastomas (*n* = 162) than in other relapsed entities. The ERBB family members were either lower (*EGFR*, *ERBB2*, *ERBB3*) or at similar levels (*ERBB4*; Fig. 4B).

As *ABCB1* is only one member of a large family of transporters, we also analyzed the expression levels of the remaining ABC family in the above datasets. In the patient dataset by Schramm et al. and all cell line datasets (Utnes, Jagannathan, Maris, Versteeg), no ABC transporter other than *ABCB1* was differentially expressed (Figs S3–S8). In the INFORM dataset, only two other transporters (*ABCA3* and *ABCG4*) were significantly (*P* < 0.05) and relevantly (FC > 2) upregulated in relapsed neuroblastoma compared to other entities (Fig. S9). Of the three, *ABCB1* had the strongest difference between the groups, with a 3.3‐fold higher expression in neuroblastoma.

Taken together, these expression datasets from neuroblastoma patient samples and cell lines point toward a major role of P‐gp specifically in neuroblastoma resistance at relapse, while the ERBB family appears to play a minor role.

### 3.4 ERBB4‐independent VCR‐sensitizing activity of afatinib

**Fig. 5:**
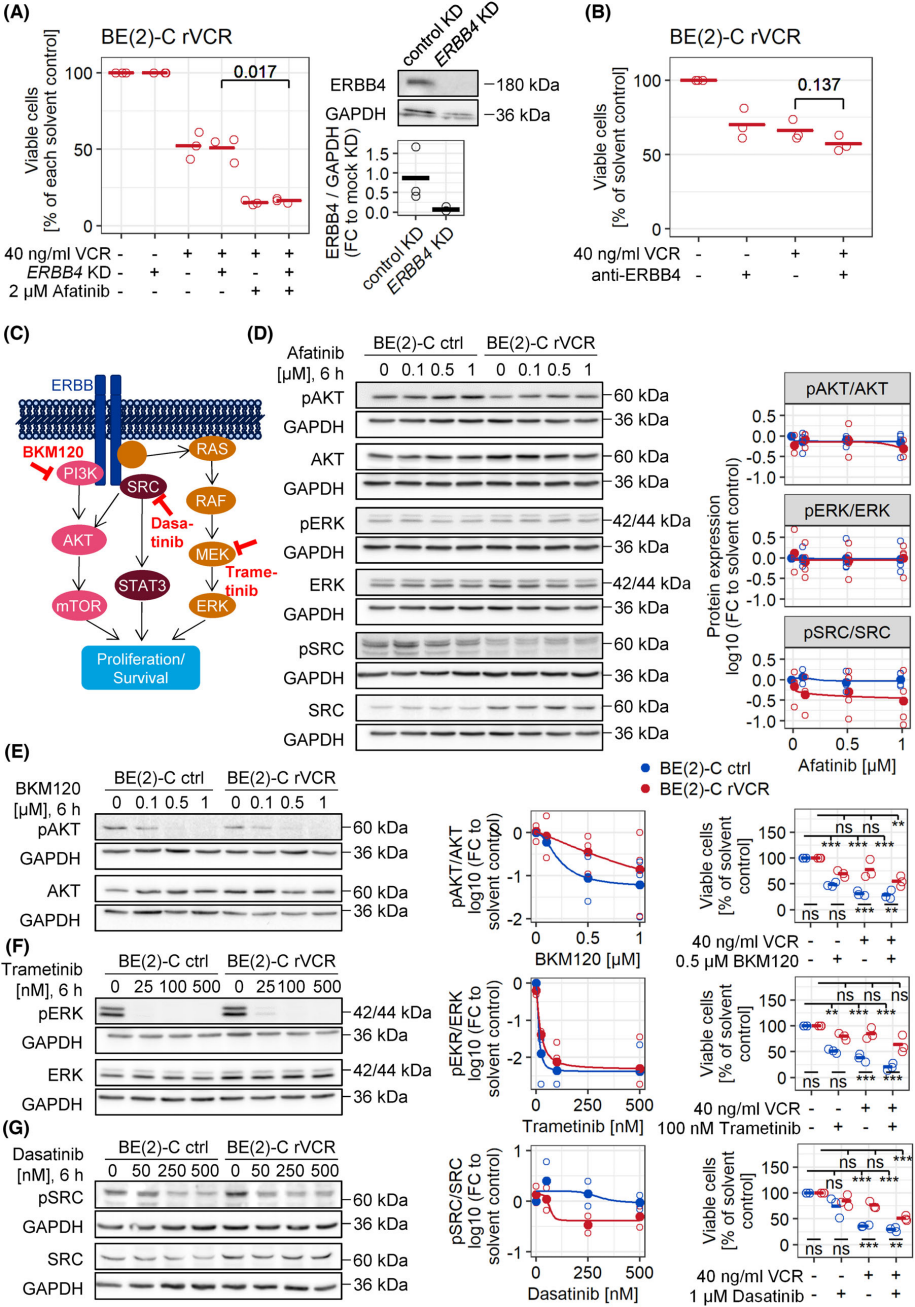
Afatinib breaks VCR resistance independent of ERBB4. (A) *ERBB4* knockdown and control transfection were performed on BE(2)‐C rVCR cells, which were treated 24 h later with 40 ng·mL^−1^ VCR and/or 2 μm afatinib for 48 h. Dead cells were stained with trypan blue, and only viable cells were counted. The percent of viable cells relative to the solvent control of each knockdown was calculated. Presented are biological replicates (*n* = 3) and their mean. Statistics were calculated with Student's *t* test. Knockdown efficiency was validated by western blot. Quantifications were normalized to GAPDH and negative control transfection. (B) BE(2)‐C rVCR cells were treated with 1 μg·mL^−1^ anti‐ERBB4 blocking antibody and/or 40 ng·mL^−1^ VCR for 48 h. Dead cells were stained with trypan blue, and only viable cells were counted. The percent of viable cells relative to the solvent control was calculated. Presented are biological replicates (*n* = 3) and their mean. Statistics were calculated with Student's *t* test. (C) Schematic of ERBB4 downstream pathways leading to cell survival and proliferation. (D) Control BE(2)‐C and BE(2)‐C rVCR were treated with 0, 0.1, 0.5, and 1 μm afatinib for 6 h. Phosphorylation of AKT, ERK, and SRC, as well as total protein levels, were assessed by western blot. Representative blots of four biological replicates are shown. Quantified phosphoprotein expression was normalized first to the respective GAPDH, then to GAPDH normalized complete protein, and finally to solvent control of control BE(2)‐C (right panel). The same GAPDH control applies for AKT/pERK and pAKT/ERK/SRC, respectively. (E–G) Control BE(2)‐C and BE(2)‐C rVCR were treated with the indicated concentrations BKM120, trametinib or dasatinib for 6 h (western blot) or 48 h in combination with 40 ng·mL^−1^ VCR (viability assay, right most panels). The phosphorylation and complete protein levels of AKT, ERK, and SRC were assessed by western blotting. Representative blots of three biological replicates are shown. Phosphoprotein expression was normalized first to the respective GAPDH, then to GAPDH normalized complete protein and finally to the solvent control of control BE(2)‐C. Quantifications are shown in the middle panels. Viability was assessed by trypan blue exclusion. The percent of viable cells relative to the solvent control of each cell line was calculated. Presented are biological replicates (*n* = 3) and their mean. Statistics were calculated with ANOVA followed by Tukey's post‐test. Shown are the comparisons between the cell lines (bottom) as well as the comparisons to solvent control for each cell line (top). ***P < 0.001, **P < 0.01, ns, not significant.

To further specify whether afatinib sensitizes cells to VCR through its direct target ERBB4, we performed *ERBB4* knockdown. Although efficient knockdown was achieved, BE(2)‐C rVCR remained as resistant to VCR as control transfection (Fig. 5A). Furthermore, *ERBB4* knockdown did not rescue the effect of afatinib on VCR treatment. Blocking ERBB4 signaling by treatment with an ERBB4 blocking antibody similarly did not influence the sensitivity of BE(2)‐C rVCR to VCR treatment (Fig. 5B).

To investigate afatinib on‐target activity, we performed western blot analysis of the three main downstream pathways of full‐length ERBB4 (Fig. 5C). The phosphorylation levels of AKT, ERK, and SRC remained unchanged in response to afatinib treatment, indicating that afatinib has no influence on downstream signaling of the ERBB family in this context (Fig. 5D). To ensure that the pathways themselves can in principle be regulated by small molecule inhibitors in these cell lines, the cells were treated with specific inhibitors for the three main downstream pathways: BKM120 (PI3K inhibition—AKT signaling), trametinib (MEK inhibition—MAPK pathway) and dasatinib (SRC inhibition—SRC signaling). All compounds effectively blocked phosphorylation within their respective pathways but did not break resistance in the same range as afatinib (Fig. 5E–G). Notably, dasatinib, which was a high hit in the original resistance‐breaking screen, was more effective in lowering viability in combination with VCR than BKM120 or trametinib but was nevertheless not as efficient as afatinib or tariquidar.

### 3.5 VCR resistance is mediated by P‐gp

**Fig. 6:**
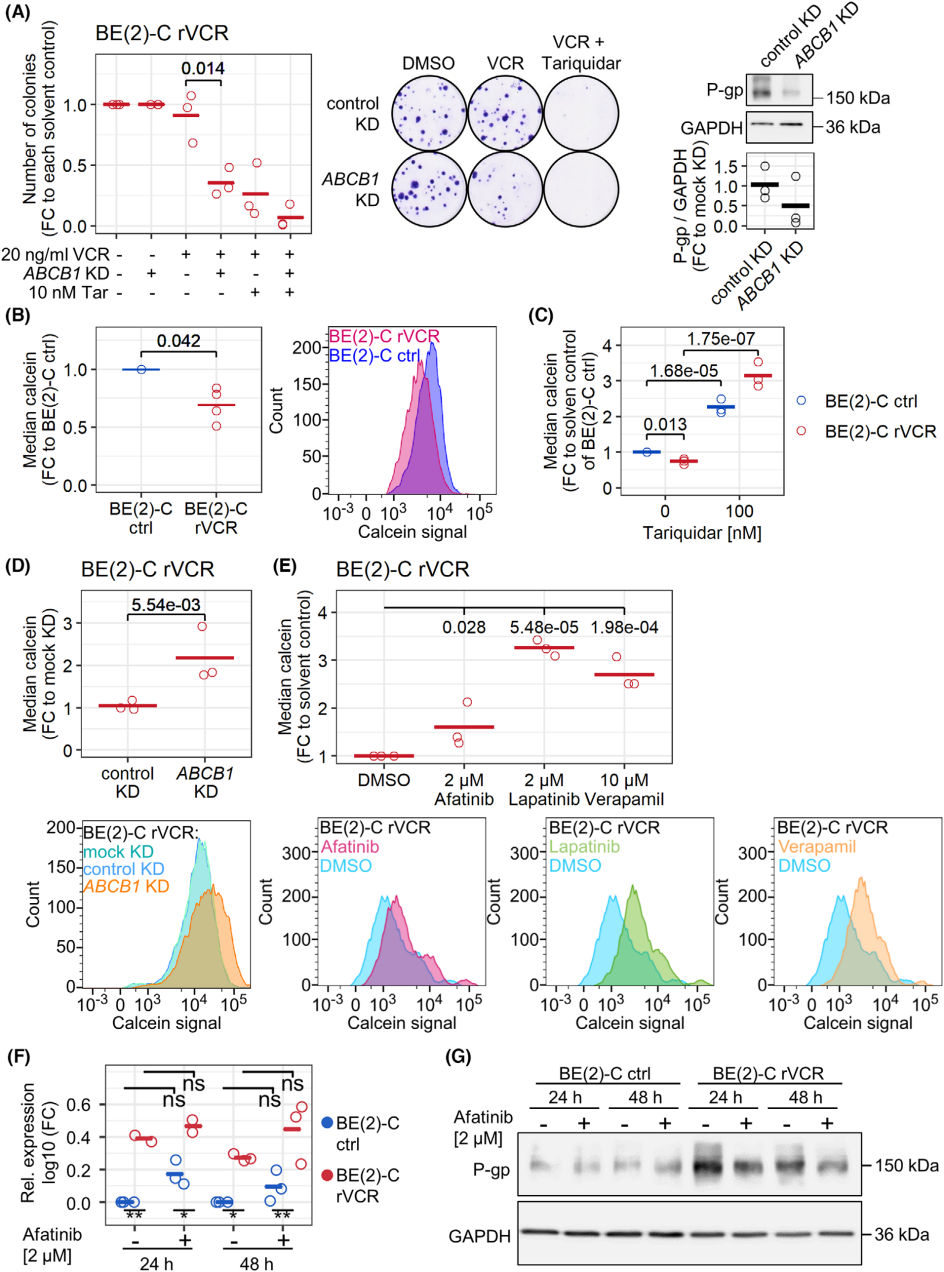
P‐gp mediates resistance in BE(2)‐C rVCR. (A) a colony assay was performed after *ABCB1* knockdown or negative control transfection. Knockdown was induced for 24 h with subsequent treatment with 20 ng·mL^−1^ VCR and/or 10 nm tariquidar for 72 h. After medium change, colonies were left to grow for 9 days. Representative example images of three biological replicates are shown. Quantification is depicted as the colony number normalized to the solvent control of each knockdown. Three biological replicates and their means are shown. Statistics of log‐transformed values were calculated with Student's *t* test. The knockdown efficiency was validated by western blotting. Quantifications were normalized to GAPDH and negative control transfection. (B) Untreated control BE(2)‐C and BE(2)‐C rVCR were stained with 10 nm calcein for 15 min and analyzed by flow cytometry. The right panel depicts a representative calcein signal distribution of four biological replicates. The median calcein signal, normalized to control BE(2)‐C, is depicted on the left. Statistics of log‐transformed BE(2)‐C rVCR values were calculated with Student's *t* test against 0. (C) Control BE(2)‐C and BE(2)‐C rVCR were treated with 100 nm tariquidar for 24 h and stained with 10 nm calcein for 15 min. Intracellular calcein was analyzed by flow cytometry. Median calcein normalized to the solvent control of BE(2)‐C ctrl is shown. Depicted are biological replicates (*n* = 3) and their mean. Statistics were computed by ANOVA of the log‐transformed values followed by Tukey's post‐test. (D) *ABCB1* knockdown and negative control transfection were performed in BE(2)‐C rVCR for a total of 48 h, followed by staining with 100 nm calcein for 30 min. Intracellular calcein was assessed by flow cytometry. The lower panel shows a representative calcein signal distribution of three biological replicates. The upper panel depicts the median calcein signal normalized to negative control transfection. Three biological replicates and their means are shown. Statistics were calculated on log‐transformed values with Student's *t*‐test. (E) BE(2)‐C rVCR cells were treated with the indicated concentrations of afatinib, lapatinib, or verapamil for 24 h and stained with 10 nm calcein for 15 min. Intracellular calcein was analyzed by flow cytometry. Median calcein was normalized to solvent control. Depicted are biological replicates (*n* = 3) and their mean. Representative calcein signal distributions are depicted in the lower panels. Statistics were computed by ANOVA of the log‐transformed values followed by Tukey's post‐test. (F, G) BE(2)‐C ctrl and rVCR were treated for 24 and 48 h with 2 μm afatinib. ABCB1 mRNA (F) and P‐gp protein (G) levels were evaluated by RT–PCR and western blotting, respectively. GAPDH served as a loading control. Statistics were calculated with ANOVA followed by Tukey's post‐test. Shown are the statistics for the comparisons between the cell lines (bottom) as well as the comparisons to solvent control for each cell line (top).

Having excluded the direct involvement of ERBB signaling in mediating VCR resistance, we further investigated the functional role of P‐gp. We performed a colony assay after *ABCB1* knockdown in BE(2)‐C rVCR, where *ABCB1* knockdown significantly decreased the ability of the cells to form colonies in the presence of VCR (Fig. 6A). Mechanistically, we suspected that elevated P‐gp levels led to increased drug efflux in BE(2)‐C rVCR cells. This was confirmed by our finding that BE(2)‐C rVCR retained less of the P‐gp substrate calcein than the control BE(2)‐C, indicating increased efflux (Fig. 6B). Treatment with the P‐gp inhibitor tariquidar greatly increased the calcein signal in both cell lines, that is, reducing the efflux (Fig. 6C). This increase in calcein signal was much stronger in BE(2)‐C rVCR compared to control BE(2)‐C. *ABCB1* knockdown in BE(2)‐C rVCR also reduced calcein efflux, thus showing the importance of P‐gp for the efflux of xenobiotics in BE(2)‐C rVCR (Fig. 6D). Treatment with the ERBB inhibitors afatinib and lapatinib as well as the P‐gp inhibitor verapamil similarly increased the calcein signal significantly (*P* = 0.28; *P* < 0.001; *P* < 0.001) in BE(2)‐C rVCR cells and directly blocked calcein efflux (Fig. 6E). To investigate the direct effects of afatinib on P‐gp expression, we analyzed the *ABCB1* mRNA and P‐gp protein levels after 24 and 48 h of afatinib treatment. Whereas *ABCB1* mRNA levels were not significantly altered, protein levels were slightly affected and decreased in the BE(2)‐C rVCR cell line but not the BE(2)‐C ctrl line (Fig. 6F,G). Similarly, we investigated the effects of downstream pathway inhibition on P‐gp expression upon 24 and 48 h of BKM120, trametinib, or dasatinib treatment. None of the inhibitors significantly affected *ABCB1* mRNA, and protein levels were slightly affected and decreased in the BE(2)‐C rVCR cell line treated with the SRC inhibitor dasatinib (Fig. S10). Hence, interference with P‐gp results in sensitization of the BE(2)‐C rVCR cell line toward VCR treatment, and afatinib also affects P‐gp function.

### 3.6 Combination treatment results in induction of apoptosis *in vitro* and decreased tumor volume *in vivo*

**Fig. 7:**
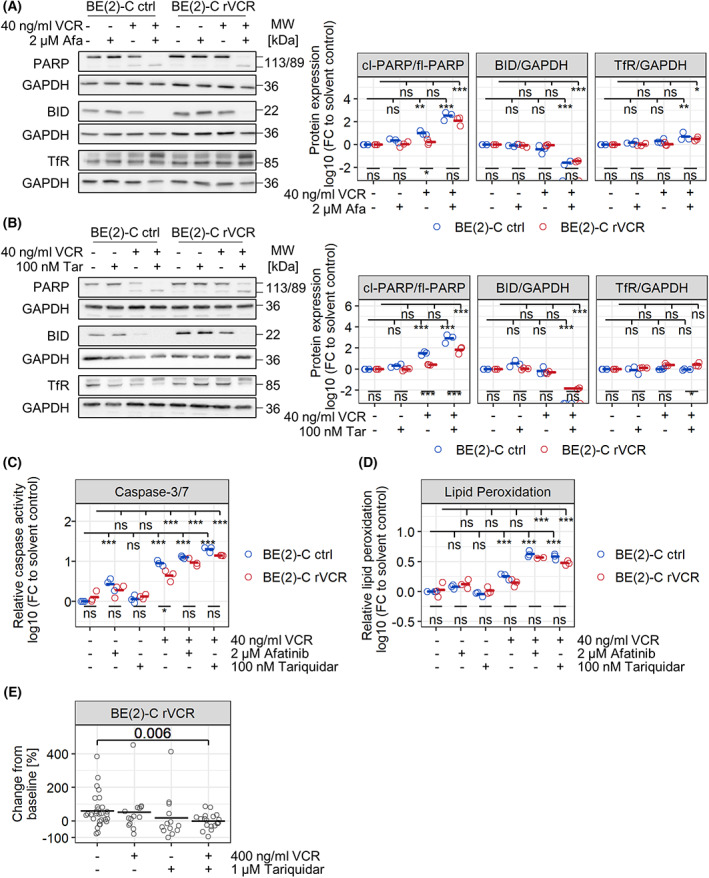
Combination of VCR with afatinib or tariquidar induces apoptosis and reduces tumor volume *in vivo*. (A–D) Control BE(2)‐C and BE(2)‐C rVCR were treated with 40 ng·mL^−1^ VCR, 2 μm afatinib, 100 nm tariquidar, or their combination for 48 h. all statistics were calculated with ANOVA of log‐transformed values followed by Tukey's post‐test. Shown are the statistics for the comparisons between the cell lines (bottom) as well as the comparisons to the solvent control for each cell line (top). (A, B) Western blot against PARP, BID, and transferrin receptor (TfR). Representative blots of three biological replicates are shown. Quantifications on the right depict biological replicates (*n* = 3) and their means. Fold changes depicted are cleaved PARP (cl‐PARP) over full‐length PARP (fl‐PARP), BID over GAPDH, and TfR over GAPDH, all normalized to the solvent control of each cell line. (C) Caspase‐3/7 activity was determined by cleavage of fluorescently labeled DEVD peptide in protein lysates. Depicted are biological replicates (*n* = 3) and their means, normalized to the solvent control of control BE(2)‐C. (D) Lipid peroxidation was evaluated by staining with 20 μm BODIPY 581/591 for 30 min followed by flow cytometric analysis of oxidized BODIPY. Depicted are biological replicates (*n* = 3) and their means normalized to the solvent control of control BE(2)‐C. (E) Change in tumor volume from day one post‐injection to day three post‐injection in zebrafish embryo xenografts with BE(2)‐C rVCR. Zebrafish embryos were treated with the indicated concentrations of VCR and tariquidar for 48 h. Each circle reflects one individual xenografts (solvent control *n* = 29; VCR *n* = 14; tariquidar *n* = 13, combo *n* = 19); means are presented by the black line. Statistics were calculated with Student's *t* test. ***P < 0.001, **P < 0.01, *P < 0.05, ns, not significant.

As the combination treatment was very effective in decreasing the viability of resistant neuroblastoma cells, we further investigated whether the combination treatment induced programmed cell death. We performed a detailed programmed cell death analysis of the combination of VCR with afatinib or tariquidar in control BE(2)‐C and BE(2)‐C rVCR. Caspase activation was demonstrated by cleavage of PARP and BID (Fig. 7A,B) and by an enzymatic activity assay (Fig. 7C). The resistance of BE(2)‐C rVCR to VCR was also detected at the level of cell death activation, by reduced levels of PARP and BID cleavage and significantly weaker caspase activation compared to control BE(2)‐C. Two markers of ferroptosis, transferrin receptor expression (Fig. 7A,B) and levels of lipid peroxidation (Fig. 7D), were only slightly elevated, indicating apoptosis to be the main method of cell death. Taken together, these analyses demonstrate that both combinations induce apoptosis.

Finally, we validated the reduction in VCR resistance by P‐gp inhibition in an *in vivo* xenograft model of zebrafish embryos. Whereas VCR single treatment was only effective in tumors derived from the BE(2)‐C ctrl line, the combination treatment of VCR with tariquidar significantly decreased the volume of BE(2)‐C rVCR‐derived tumors in zebrafish embryos (Figs 7E and S11).

## 4 Discussion

Chemoresistance is a major factor limiting treatment success in relapsed neuroblastomas. Here, we investigated how this chemotherapy resistance can be overcome by studying VCR‐resistant sublines of the high‐risk neuroblastoma cell lines SK‐N‐BE(2)‐C, MHH‐NB‐11, NGP, and NB‐S‐124. We showed with the SK‐N‐BE(2)‐C‐derived model that resistance to VCR was most efficiently reversed by the ERBB family inhibitors afatinib and lapatinib, as well as the P‐gp inhibitors tariquidar and verapamil.

In cell‐free assays, the IC50 of afatinib was determined to be 0.5 nm for EGFR, 1 nm for ERBB4, and 14 nm for ERBB2 [43], and the IC50 for lapatinib was determined to be 10 nm for EGFR, 9 nm for ERBB2, and 367 nm for ERBB4 [44]. Unlike many other ERBB family inhibitors, such as erlotinib and gefitinib, afatinib, and lapatinib are thus able to target ERBB4 with high affinity, which was the only one of the three main targets of afatinib to be upregulated in BE(2)‐C‐rVCR. ERBB3, which was also upregulated at the mRNA level, is kinase impaired, and its limited phosphorylation capacity is not suppressed by ERBB family inhibitors [15].

Despite its capability to efficiently break VCR resistance, afatinib did not influence any of the three main downstream pathways of full‐length ERBB4, nor did ERBB4 knockdown influence VCR resistance. Direct inhibition of the three main downstream pathways of ERBB4 by BKM120, trametinib, and dasatinib showed no or minimal reduction in VCR resistance at concentrations that efficiently reduced phosphorylation of key proteins in the respective pathways. Dasatinib showed the most promising, albeit incomplete, resistance‐breaking effect when used at the highest concentration of the metabolic activity screen.

In addition to their effect on the ERBB family, afatinib and lapatinib are also known substrates of P‐gp and have been shown to break chemotherapy resistance by competitively inhibiting P‐gp [45, 46]. If afatinib is transported out of the cells directly and efficiently via P‐gp, this might explain the lack of effect of afatinib on ERBB4 downstream signaling pathways. Moreover, competitive inhibition blocks P‐gp and subsequently elevates the intracellular VCR concentrations, effectively killing the cells. Lapatinib might sensitize the cells through the same mechanism. Trametinib and dasatinib are also known to be P‐gp substrates [47, 48] but do not overcome resistance. As both trametinib [48] and dasatinib [49, 50] inhibit P‐gp at concentrations > 5 μm, the chosen, target‐specific concentrations (≤ 1 μm) were too low for the resistance‐breaking effect observed with afatinib. Indeed, at 1 μm, dasatinib started to show an effect on viability when combined with VCR. BKM120 is not a substrate of P‐gp and accordingly did not attenuate P‐gp resistance [51].

Although all BE(2)‐C‐derived models expressed extraordinarily high levels, *ABCB1*/P‐gp itself was not significantly upregulated at the mRNA level in BE(2)‐C rVCR compared to control BE(2)‐C. Furthermore, P‐gp is not exclusively found at the plasma membrane but also in intracellular compartments such as the endoplasmic reticulum, Golgi apparatus or lysosomes [52, 53], so increased accumulation at the plasma membrane might increase drug efflux. Indeed, surface P‐gp was significantly increased in BE(2)‐C rVCR. Functional assays using calcein efflux further showed that P‐gp function is upregulated in BE(2)‐C rVCR. In line with the described effects on P‐gp, the P‐gp inhibitors tariquidar and verapamil, as well as *ABCB1* knockdown, were very efficient in sensitizing cells to VCR treatment.

Moreover, a connection between ERBB signaling and *ABCB1* expression has been described in ovarian and lung cancer models: Afatinib decreased *ABCB1* expression in *ABCB1*‐overexpressing cells by inhibiting the activation of PI3K/AKT signaling and NF‐κB activation [46, 54]. In our study on neuroblastoma cells, however, we could not find evidence of AKT signaling being altered upon afatinib treatment. Hence, in our model system, it is more likely that all four inhibitors—the ERBB inhibitors afatinib and lapatinib and the P‐gp inhibitors tariquidar and verapamil—act in a P‐gp‐dependent manner in overcoming VCR resistance.

P‐gp is only one member of a large family of transporter proteins, several others of which contribute to multiple drug resistance in cancer. Afatinib is also known to be transported by ABCC1 and ABCG2 [55, 56]. However, while *ABCB1* is consistently upregulated in several cohorts of neuroblastoma cell lines and patient samples, other members of the ABC family are not regulated. This finding points to a relevant role of P‐gp in neuroblastoma.

## 5 Conclusions

In summary, we investigated chemotherapy resistance through VCR‐resistant sublines of high‐risk neuroblastoma cell lines. We effectively overcame the resistance of the SK‐N‐BE(2)‐C‐derived model *in vitro* and *in vivo* by the addition of the pan‐ERBB family inhibitors afatinib and lapatinib, as well as the P‐gp inhibitors tariquidar and verapamil, through the induction of apoptosis. As we found no evidence of the involvement of ERBB signaling, we conclude that the resistance‐breaking effect observed is due to competitive inhibition of P‐gp and increased presence of VCR in the cells. The analysis of gene expression datasets of more than 50 different neuroblastoma cell lines (primary and relapsed) and more than 160 neuroblastoma patient samples from the pediatric precision medicine platform INFORM (Individualized Therapy For Relapsed Malignancies in Childhood) confirmed a pivotal role of P‐gp specifically in neuroblastoma resistance at relapse, while the ERBB family appears to play a minor part. This study underlines the importance of target validation (off‐target effects of ERBBi) and of repurposing clinically approved drugs.

## Conflict of interest

Olaf Witt participated in the advisory boards of Novartis, BMS, Janssen, and receives research grants from BVD, Day One Therapeutics.

## Author contributions

HP and IO conceived and designed the project; LR, SH, and SN acquired the data; LR, SH, SN, JR, HP, and MM provided methods and protocols; LR and SN validated the results; LR analyzed and interpreted the data; LR visualized the results; JC, DTWJ, MM, OW, and IO provided resources; JC, DTWJ, and MM, provided interpretation of data and revised the work critically for scientific content; LR and IO stored and managed the data; LR and IO wrote the original draft; LR, SH, JR, HP, JC, DTWJ, MM, OW, and IO reviewed and edited the manuscript; OW and IO supervised the project; IO acquired funding. All authors have read and agreed to the published version of the manuscript.

## Supporting information


**Fig. S1.** Combining VCR with afatinib or tariquidar results in a synergistic reduction in viability.
**Fig. S2.** Expression of ERBB family and ABCB1 in R2 datasets.
**Fig. S3.** Expression of the ABC gene family in gene expression data of paired samples of patients at primary diagnosis and at relapse.
**Fig. S4.** Expression of the ABC gene family in gene expression data of paired cell lines (Utnes et al.) derived at primary diagnosis and at relapse.
**Fig. S5.** Expression of the ABC gene family in gene expression data of cell lines (Jagannathan et al.) derived at primary diagnosis and at relapse.
**Fig. S6.** Expression of the ABC gene family in gene expression data of cell lines (Maris et al.) derived at primary diagnosis and at relapse.
**Fig. S7.** Expression of the ABC gene family in gene expression data of cell lines (Versteeg et al.) derived at primary diagnosis and at relapse.
**Fig. S8.** Expression of the ABC gene family in gene expression data of cell lines (Broad Institute) derived at primary diagnosis and at relapse.
**Fig. S9.** Expression of the ABC gene family in gene expression data of relapsed neuroblastoma compared to other relapsed pediatric tumor entities.
**Fig. S10.** ABCB1/P‐gp expression upon downstream pathway inhibition.
**Fig. S11.** Zebrafish embryo xenograft model.

## Data Availability

Supporting data are available as Supplementary Information.
