# Supplementary material for: ERBB and P‐glycoprotein inhibitors break resistance in relapsed neuroblastoma models through P‐glycoprotein
Source: Mol Oncol. 2022 Nov 5;17(1):37–58. doi: 10.1002/1878-0261.13318 (PMC9812835; doi:10.1002/1878-0261.13318)
Supplement: Supplementary file 1 — Fig. S1. Combining VCR with afatinib or tariquidar results in a synergistic reduction in viability. Fig. S2. Expression of ERBB family and ABCB1 in R2 datasets. Fig. S3. Expression of the ABC gene family in gene expression data of paired samples of patients at primary diagnosis and at relapse. Fig. S4. Expression of the ABC gene family in gene expression data of paired cell lines (Utnes et al.) derived at primary diagnosis and at relapse. Fig. S5. Expression of the ABC gene family in gene expression data of cell lines (Jagannathan et al.) derived at primary diagnosis and at relapse. Fig. S6. Expression of the ABC gene family in gene expression data of cell lines (Maris et al.) derived at primary diagnosis and at relapse. Fig. S7. Expression of the ABC gene family in gene expression data of cell lines (Versteeg et al.) derived at primary diagnosis and at relapse. Fig. S8. Expression of the ABC gene family in gene expression data of cell lines (Broad Institute) derived at primary diagnosis and at relapse. Fig. S9. Expression of the ABC gene family in gene expression data of relapsed neuroblastoma compared to other relapsed pediatric tumor entities. Fig. S10. ABCB1/P‐gp expression upon downstream pathway inhibition. Fig. S11. Zebrafish embryo xenograft model. [file MOL2-17-37-s001.pdf]

ERBB and P-glycoprotein inhibitors break resistance in relapsed neuroblastoma models through P-glycoprotein

Lisa Rösch [1,2,3], Sonja Herter [1,2,3], Sara Najafi [1,2,4], Johannes Ridinger [1,2], Heike Peterziel [1,2], Jindrich Cinatl [5], David T. W. Jones [1,6], Martin Michaelis [7], Olaf Witt [1,2,4] and Ina Oehme [1,2,*]

1 Hopp Children's Cancer Center Heidelberg (KiTZ), Heidelberg, Germany.
2 Clinical Cooperation Unit Pediatric Oncology, German Cancer Research Center (DKFZ) and German Cancer Consortium (DKTK), Heidelberg, Germany.
3 Faculty of Biosciences, University of Heidelberg, Heidelberg, Germany.
4 Department of Pediatric Oncology, Hematology and Immunology, University Hospital Heidelberg, Heidelberg, Germany.
5 Institute for Medical Virology, Goethe University Hospital, Frankfurt am Main, Germany.
6 Division of Pediatric Glioma Research, German Cancer Research Center (DKFZ), Heidelberg, Germany.
7 School of Biosciences, University of Kent, Canterbury, United Kingdom.
* Correspondence: PD Dr. Ina Oehme, KiTZ/DKFZ, Im Neuenheimer Feld 280, 69120 Heidelberg, Germany; phone: 0049-6221-423388; i.oehme@kitz-heidelberg.de

Supplementary Information

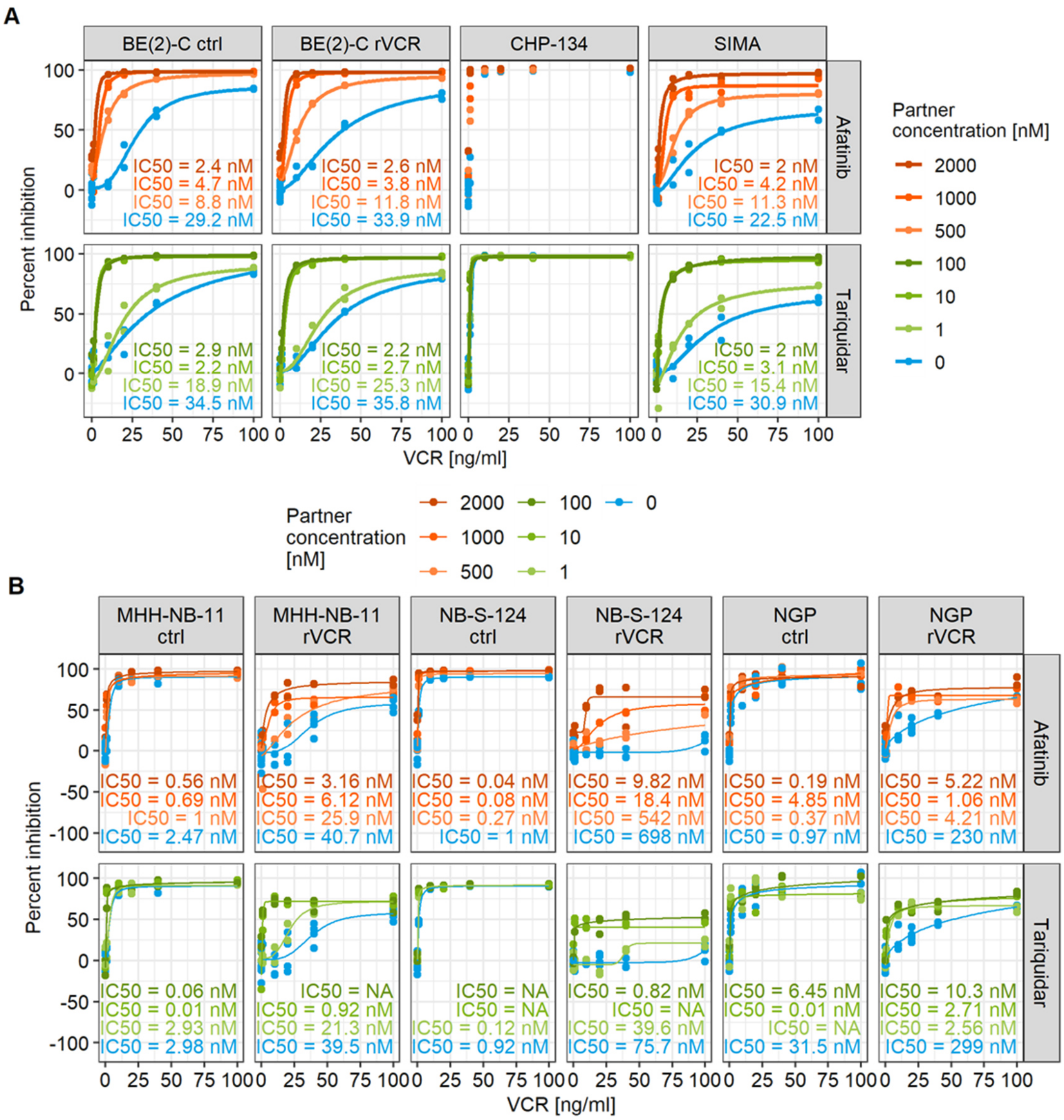

Figure S1: Combining VCR with afatinib or tariquidar results in a synergistic reduction in viability. **(A-B)** Cells were treated with 0, 10, 20, 40, or 100 ng/ml VCR +/- 0, 0.5, 1, or 2 μM afatinib or 0, 1, 10, or 1000 nM tariquidar for 48 h. Metabolic activity was assessed by CellTiter-Glo, and percent inhibition was calculated relative to controls (1 μM staurosporine and DMSO). IC50s were calculated with an LL4 model. NA = no IC50 could be computed.

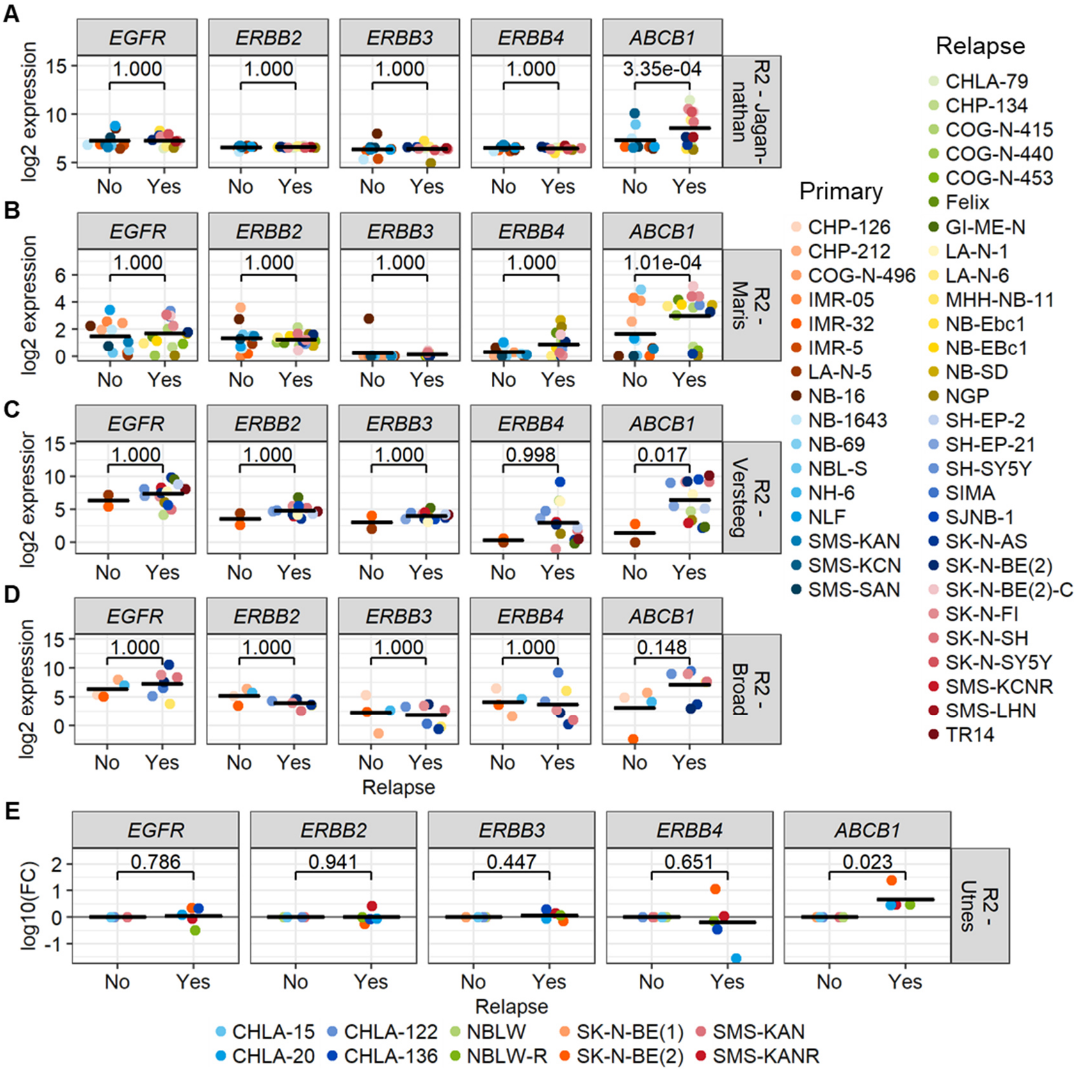

Figure S2: Expression of ERBB family and ABCB1 in R2 datasets. **(A)** Gene expression data of 38 neuroblastoma cell lines from the dataset by Jagannathan et al. (GSE19274) [36] were downloaded from R2 (08 June 2021). All cell lines that could not be positively identified as having been derived before (n = 10) or after (n = 12) the patient had received chemotherapy (Relapse No/Yes) were excluded from the analysis. **(B)** Gene expression data of 41 neuroblastoma cell lines from the dataset by Maris et al. (GSE89413) [37] were downloaded from R2 (07 January 2021). All cell lines that could not be positively identified as having been derived before (n = 12) or after (n = 15) the patient had received chemotherapy (Relapse No/Yes) were excluded from the analysis. **(C)** Gene expression data of 24 neuroblastoma cell lines from the dataset by Versteeg et al. (GSE28019) were downloaded from R2 (January 7, 2021). All cell lines that could not be positively identified as having been derived before (n = 2) or after (n = 14) the patient had received chemotherapy (Relapse No/Yes) were excluded from the analysis. **(D)** Gene expression data of 917 cell lines from the cell line encyclopedia dataset from the Broad Institute (GSE19274) [36], which included 17 neuroblastoma cell lines, were downloaded from R2 (08 June 2021). Neuroblastoma cell lines that could not be positively identified as having been derived before (n = 4) or after (n = 7) the patient had received chemotherapy (Relapse No/Yes) were excluded from the analysis. **(A-D)** Statistics were calculated by ANOVA followed by Tukey's posttest including all genes of the ERBB and ABC families present in the respective datasets. **(E)** Gene expression data of 10 paired neuroblastoma cell lines from the dataset by Utnes et al. (GSE148700) [35] were downloaded from R2 (07 January 2021). This dataset consists of five cell line pairs derived from the same patients at primary diagnosis and after relapse. Matched pairs are indicated by color. Statistics of log-transformed relapse values were calculated with Student's t test against 0.

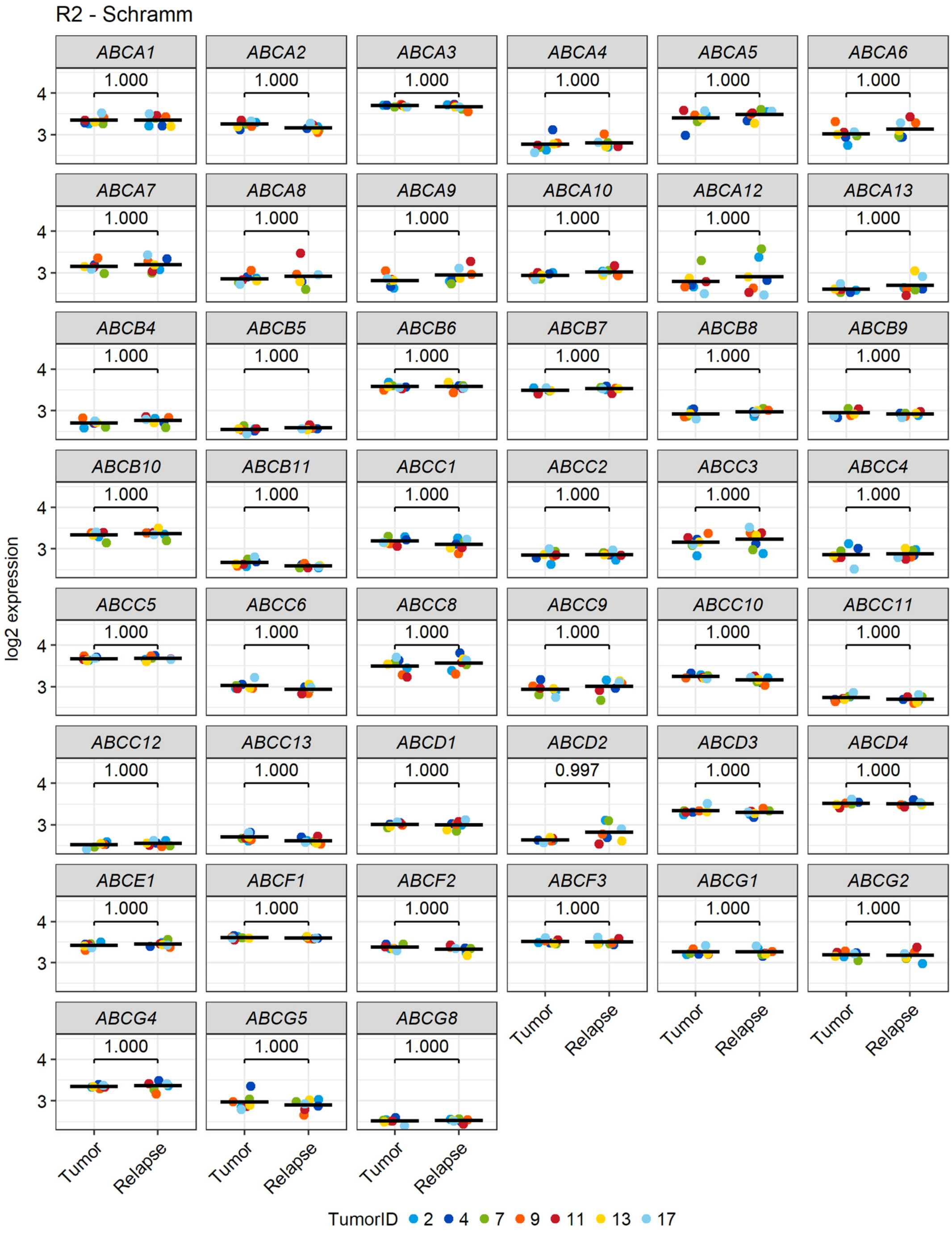

Figure S3: Expression of the ABC gene family in gene expression data of paired samples of patients at primary diagnosis and at relapse. Gene expression data of 18 tumor samples from the dataset by Schramm et al. (GSE65303) [39] were downloaded from R2 (http://r2.amc.nl, 09 September 2021). Only paired samples with data at primary diagnosis (tumor) and at relapse were included (n = 7 pairs). Statistics were calculated by ANOVA followed by Tukey's posttest including all genes of the ERBB and ABC families present in the dataset.

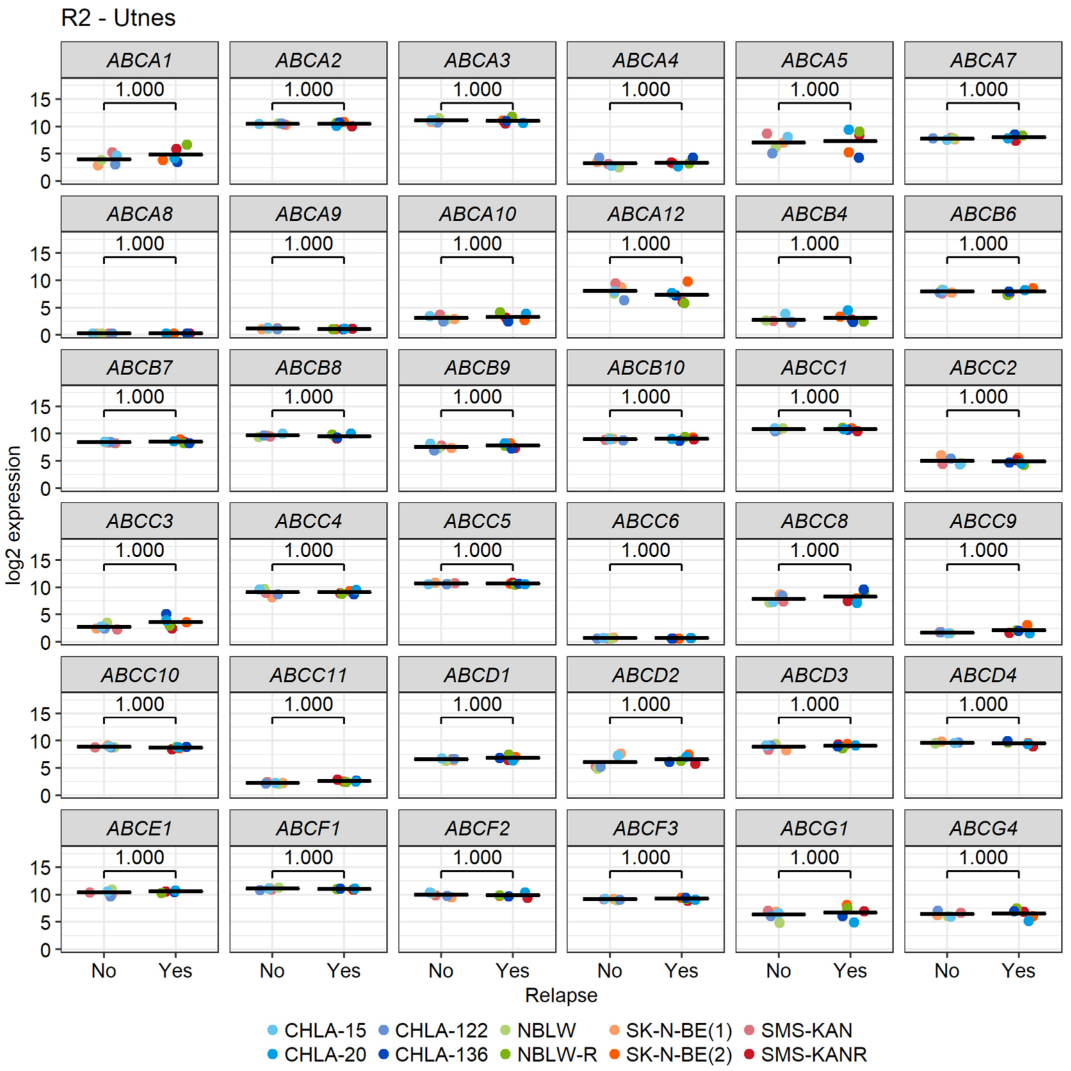

Figure S4: Expression of the ABC gene family in gene expression data of paired cell lines (Utnes et al.) derived at primary diagnosis and at relapse. Gene expression data of 10 paired neuroblastoma cell lines from the dataset by Utnes et al. (GSE148700) [35] were downloaded from R2 (http://r2.amc.nl, 07 January 2021). This dataset is made up of five cell line pairs derived from the same patients at primary diagnosis and after relapse. Matched pairs are indicated by color. Statistics were calculated by ANOVA followed by Tukey's posttest including all genes of the ERBB and ABC families present in the dataset.

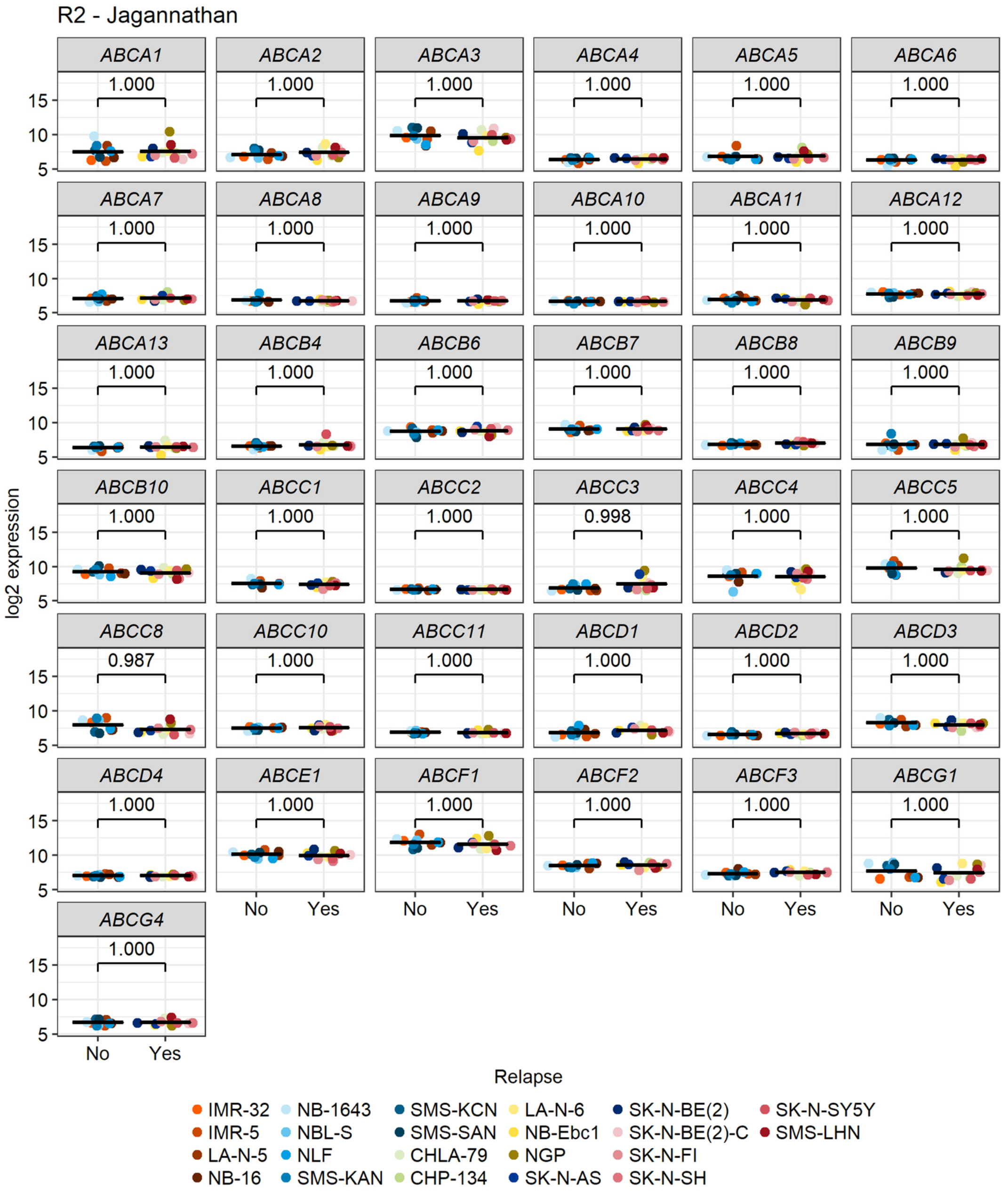

Figure S5: Expression of the ABC gene family in gene expression data of cell lines (Jagannathan et al.) derived at primary diagnosis and at relapse. Gene expression data of 38 neuroblastoma cell lines from the dataset by Jagannathan et al. (GSE19274) [36] were downloaded from R2 (http://r2.amc.nl, 08 June 2021). All cell lines that could not be positively identified as having been derived before (n = 10) or after (n = 12) the patient had received chemotherapy (Relapse No/Yes) were excluded from the analysis. Statistics were calculated by ANOVA followed by Tukey's posttest including all genes of the ERBB and ABC families present in the dataset.

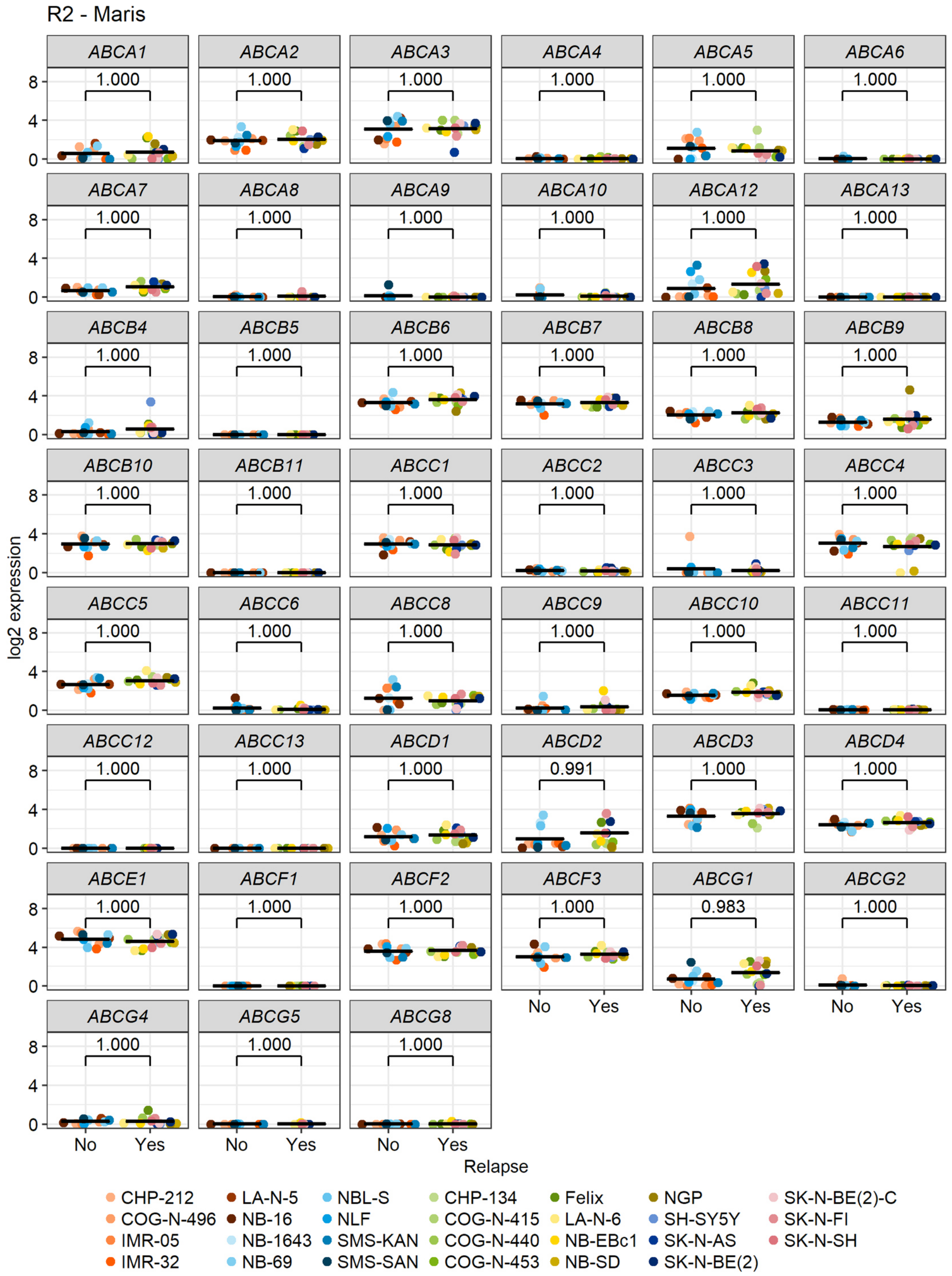

Figure S6: Expression of the ABC gene family in gene expression data of cell lines (Maris et al.) derived at primary diagnosis and at relapse. Gene expression data of 41 neuroblastoma cell lines from the dataset by Maris et al. (GSE89413) [37] were downloaded from R2 (http://r2.amc.nl, 07 January 2021). All cell lines that could not be positively identified as having been derived before (n = 12) or after (n = 15) the patient had received chemotherapy (Relapse No/Yes) were excluded from the analysis. Statistics were calculated by ANOVA followed by Tukey's posttest including all genes of the ERBB and ABC families present in the dataset.

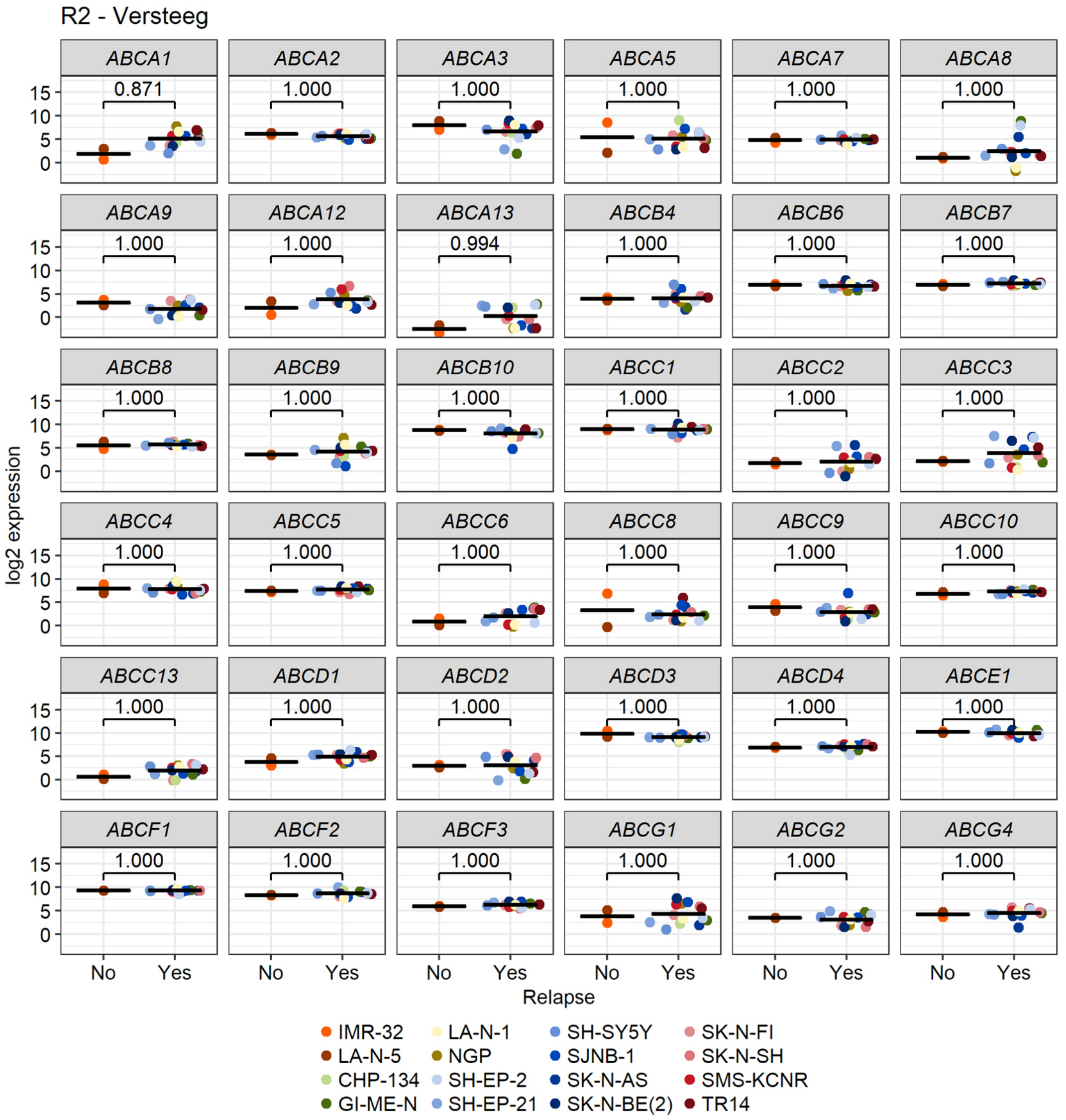

Figure S7: Expression of the ABC gene family in gene expression data of cell lines (Versteeg et al.) derived at primary diagnosis and at relapse. Gene expression data of 24 neuroblastoma cell lines from the dataset by Versteeg et al. (GSE28019) were downloaded from R2 (http://r2.amc.nl, 07 January 2021). All cell lines that could not be positively identified as having been derived before (n = 2) or after (n = 14) the patient had received chemotherapy (Relapse No/Yes) were excluded from the analysis. Statistics were calculated by ANOVA followed by Tukey's posttest including all genes of the ERBB and ABC families present in the dataset.

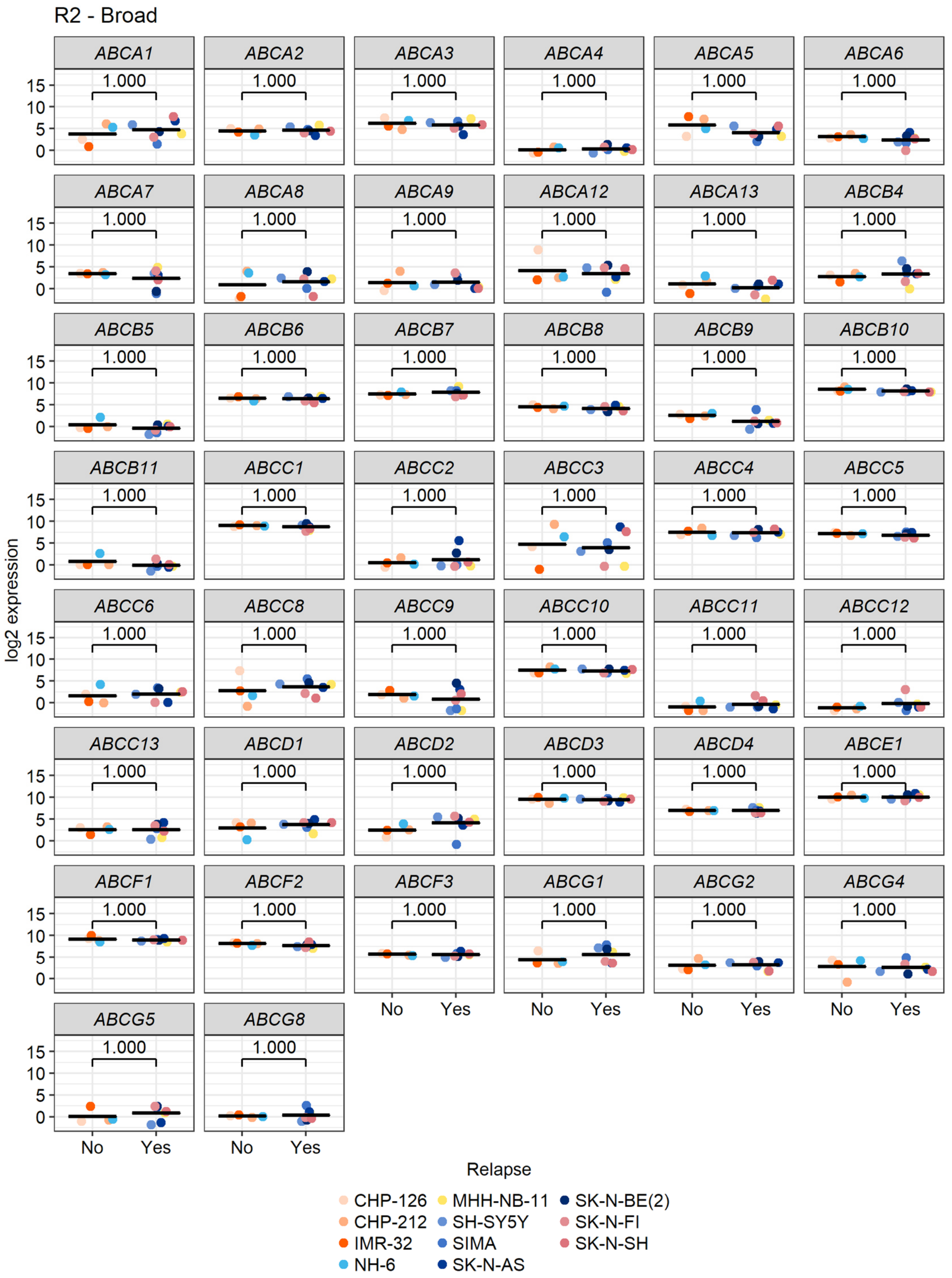

Figure S8: Expression of the ABC gene family in gene expression data of cell lines (Broad Institute) derived at primary diagnosis and at relapse. Gene expression data of 917 cell lines from the cell line encyclopedia dataset from the Broad Institute (GSE19274) [38], which comprised 17 neuroblastoma cell lines, were downloaded from R2 (http://r2.amc.nl, 08 June 2021). All cell lines that could not be positively identified as having been derived before ($n = 4$) or after ($n = 7$) the patient had received chemotherapy (Relapse No/Yes) were excluded from the analysis. Statistics were calculated by ANOVA followed by Tukey's posttest including all genes of the ERBB and ABC families present in the dataset.

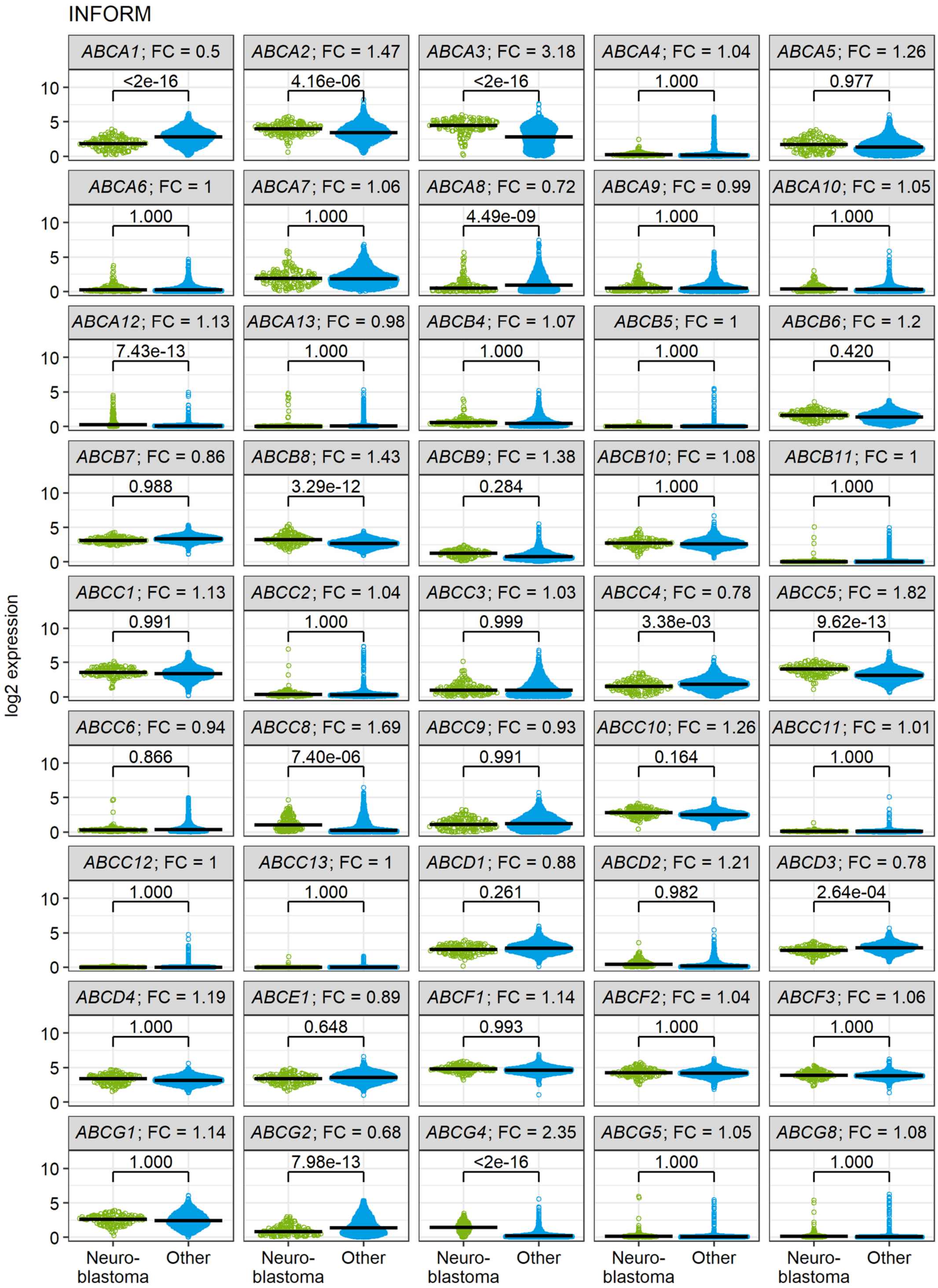

Figure S9: Expression of the ABC gene family in gene expression data of relapsed neuroblastoma compared to other relapsed pediatric tumor entities. Gene expression data from the INFORM registry (*n* =1645, [40,41]) were downloaded from R2 (http://r2.amc.nl). Comparison of neuroblastoma cases (*n* = 162) vs. all other entities. Fold change between the group means is indicated in the panel titles. Statistics were calculated by ANOVA followed by Tukey's posttest including all genes of the ERBB and ABC families present in the dataset.

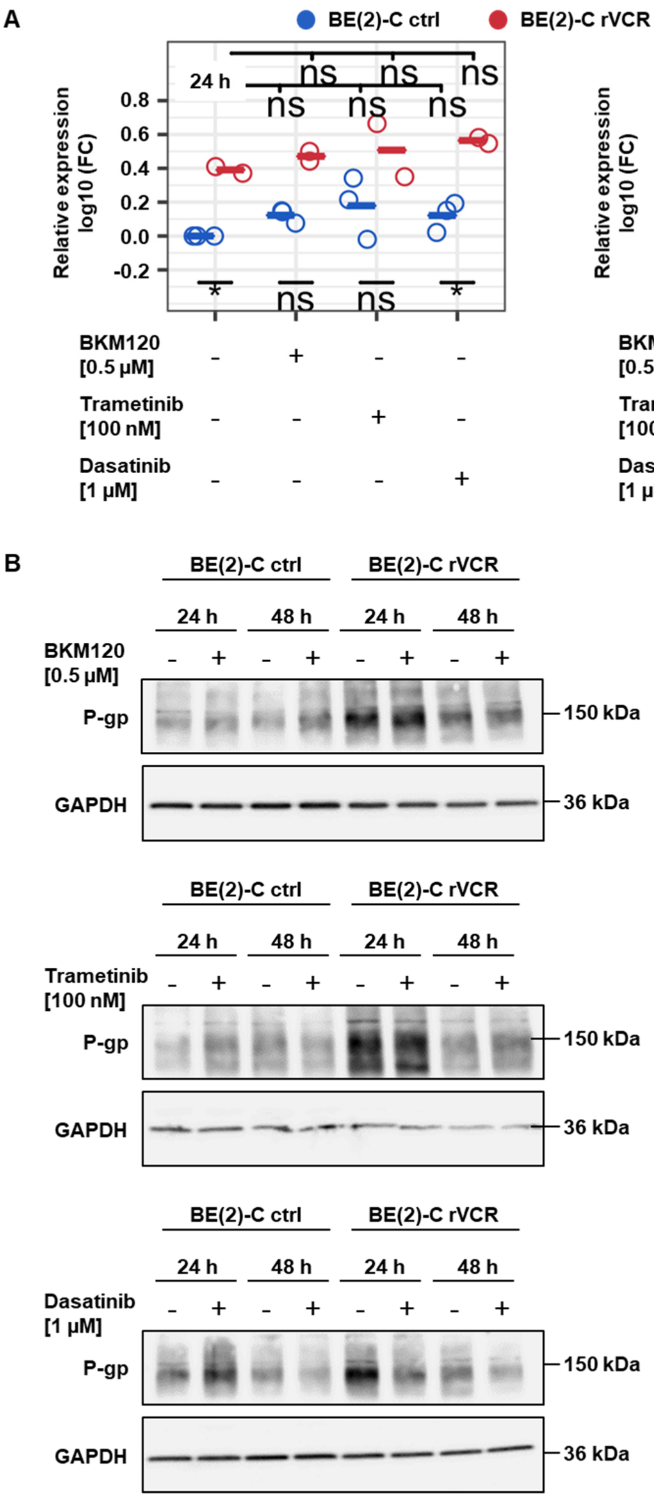

Figure S10: ABCB1/P-gp expression upon downstream pathway inhibition. **(A)** Real-time PCR of ABCB1 was performed on cDNA of control BE(2)-C and BE(2)-C rVCR treated with the indicated concentrations of BKM120, trametinib or dasatinib for 24 h (left panel) or 48 h (right panel). Presented are biological replicates and their mean. Statistics were calculated with ANOVA followed by Tukey's posttest. Shown are the comparisons between the cell lines (bottom) as well as the comparisons to solvent control for each cell line (top). **(B)** Control BE(2)-C and BE(2)-C rVCR cells were treated with the indicated concentrations of BKM120, trametinib or dasatinib for 24 h or 48 h. Complete protein levels of P-gp were assessed by western blot. Representative blots of three biological replicates are shown.

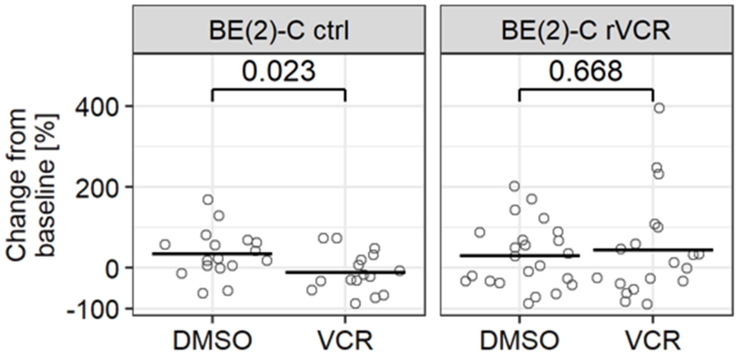

Figure S11: Zebrafish embryo xenograft model. Change in tumor volume from day one post injection to day three post injection in zebrafish embryo xenografts with BE(2)-C ctrl and rVCR, respectively, as indicated. Zebrafish embryos were treated with 400 ng/ml VCR for 48 h. Individual xenografts (circles) and their means (lines) are presented. Group sizes: solvent control BE(2)-C ctrl n=17; VCR BE(2)-C ctrl n=16; solvent control BE(2)-C rVCR n=23; VCR BE(2)-CrVCRctrl n=19. Statistics were calculated with Student's t test, and p values are indicated.
